# Trait‐mediated filtering of *Phytophthora* pathogen invasions through global horticultural trade networks

**DOI:** 10.1111/nph.70587

**Published:** 2025-09-19

**Authors:** Louise J. Barwell, Bethan V. Purse, Sarah Green, Giles Hardy, Peter Scott, Nari Williams, David E. L. Cooke, Ana Perez‐Sierra, Treena I. Burgess, Daniel Chapman

**Affiliations:** ^1^ UK Centre for Ecology and Hydrology Maclean Building, Benson Lane Wallingford OX10 8BB UK; ^2^ Forest Research Northern Research Station Roslin Midlothian EH25 9SY UK; ^3^ ArborCarbon Pty Ltd ROTA Trans 1 Murdoch University Murdoch WA 6150 Australia; ^4^ Plant & Food Research Cnr Crosses and St George's Roads Havelock North 4130 New Zealand; ^5^ The James Hutton Institute Invergowrie Dundee DD2 5DA UK; ^6^ Forest Research Alice Holt Lodge Farnham Surrey GU10 4LH UK; ^7^ Phytophthora Science and Management, Harry Butler Institute Murdoch University Murdoch 6150 WA Australia; ^8^ Biological and Environmental Sciences University of Stirling Stirling FK9 4LA UK

**Keywords:** biological invasions, *Phytophthora*, plant health, prioritisation, risk assessment, surveillance, traits

## Abstract

- Estimates of invasion risk can support prioritisation of future threats from non‐native species. Greater risk of invasion is expected when species occur in connected source regions and possess traits promoting successful transport, introduction or establishment.
- We compile a global database of first reports of *Phytophthora* de Bary species, a diverse oomycete genus attacking a broad range of plant hosts across multiple regions, sectors and ecosystem types with increasing frequency. Using Bayesian hierarchical zero‐inflated models, we model global patterns of new detections since 2005 among 109 *Phytophthora* pathogens across 56 countries with at least two known *Phytophthora* species reported before 2005. We estimate the effects of trade connectivity, climate matching, national surveillance and pathogen traits on the probability of a new detection.
- We find that 69 (38%) *Phytophthora* species were either unknown or had no known source regions before 2005 and were therefore excluded from our analysis. Our study shows that invasion risk is increased for pathogens with broader thermal tolerance and the ability to produce survival structures linked to stress tolerance and asymptomatic infections.
- This knowledge can be used to enhance national horizon scanning and risk‐based surveillance activities to better manage risks to plant health from emerging pathogens.

## Introduction

Invasive plant pests and pathogens impose significant economic costs on forestry, horticulture and agriculture, and threaten the biodiversity and ecosystem benefits of forests and urban trees (Seidl *et al*., 2018; Fei *et al*., 2019; Hill *et al*., 2019; Tabassum *et al*., 2024). Global trade networks continue to diversify, connecting previously isolated biogeographic regions (Banks *et al*., 2015; Seebens *et al*., 2018). Among alien plant species in Europe, over 80% of invasive species and 70% of naturalised species were originally introduced via trade in ornamental horticulture because of the sourcing of plants‐for‐planting via import (van Kleunen *et al*., 2018). The horticultural pathway facilitates not just plant invasions but the co‐introduction of pests and pathogens (Hulme *et al*., 2008; Roy *et al*., 2014; Dehnen‐Schmutz, 2010). Import volumes are strong predictors of temporal trends in the emergence of novel pests and diseases of forests (Sikes *et al*., 2018; MacLachlan *et al*., 2021). It is estimated that *c*. 70% of invasive pests and pathogens established in US forests since 1860 were introduced with imported plants (Liebhold *et al*., 2012) and variability in commercial demand for trees has been linked to the risk of novel pathogen introductions (Alonso Chavez *et al*., 2019).

International trade in horticultural plants is predicted to continue to increase in Europe and North America, and to accelerate in south, central and eastern Asia (van Kleunen *et al*., 2018). In addition, tree planting and restoration initiatives not only play a central role in climate change mitigation policies and efforts to meet agreed targets for biodiversity and well‐being (Bastin *et al*., 2019; Saint Laurent *et al*., 2019; Doelman *et al*., 2020) but also may create new pathways of introduction of non‐native pests and diseases if biosecurity is lacking in the supply chains producing trees for planting (Mitchell *et al*., 2024).

The biological invasion process consists of multiple stages, beginning with entrainment in viable pathways, followed by transport, introduction, establishment, spread and subsequent ecological and socio‐economic impacts (Blackburn *et al*., 2011, 2014). Following transport, some pathways of onward spread involve diverse actors and can only be poorly understood and monitored. The ultimate destinations of traded plants for planting span managed and natural ecosystems, including public and private gardens, forestry plantations, urban and amenity plantings and restoration projects (Alonso Chavez *et al*., 2019; Rooney‐Latham *et al*., 2019). The impacts of plant diseases are often too severe for mitigation to be cost‐effective by the time that awareness and willingness to intervene have grown (Jones & Kleczkowski, 2020). It follows that strategic prioritisation of high‐risk pathogens to enhance early detection and rapid response has the greatest potential to improve outcomes for plant health (Brasier, 2008; White *et al*., 2019), particularly for native and commercial forests, which have experienced significant damage linked to the accumulation of non‐native pests and diseases in recent decades (Bonello *et al*., 2020; de Groot, 2020).

Pathogen invasion risk is proportional to propagule pressure (Cassey *et al*., 2018; MacLachlan *et al*., 2021), which refers to the quantity and frequency with which individuals are introduced to a location. Useful proxies for propagule pressure can be derived by identifying and quantifying trade flows in commodities that are viable pathways for transport (Sikes *et al*., 2018). Integrating bilateral trade networks and bioclimatic similarity (to account for post‐transport establishment risk) between source regions and importing countries has proved especially informative about recent invasions of pests in the European and Mediterranean basin region (D. Chapman *et al*., 2017). However, biological characteristics of pathogens may also explain interspecific differences in invasion success by influencing pretransport exposure to trade pathways (entrainment), responses to environmental conditions during transport, establishment and spread, and ability to evade surveillance. Pathogen traits are identified as a key knowledge gap in understanding the emergence of forest diseases (Guégan *et al*., 2023), and a number of traits have been proposed as potentially important predictors of establishment and spread. These include spore size, optimum temperature for growth, the abilities to disperse long distances, to reproduce both sexually and asexually (Philibert *et al*., 2011), to attack both forest and horticultural hosts (Santini *et al*., 2013), as well as cold‐tolerance and asexual survival structures (Redondo *et al*., 2018). Species filtering during transport may be particularly extreme (Briski *et al*., 2018), yet few studies have tested for trait‐mediated effects on transport success of plant pathogens. For parasites of nonplant hosts, their distribution (widespread or local) and host‐use (prevalence, generalism, capacity to infect traded commodities) within the source region have been proposed as useful predictors of translocation to new regions (MacLeod *et al*., 2010; Ewen *et al*., 2012; Blackburn & Ewen, 2017). These data are often unavailable for emerging plant pathogens because their diversity, distributions and hosts are severely under‐reported. Indeed, many high‐profile forest pathogens were not described until they emerged in a novel region (e.g. *Phytophthora ramorum*). By contrast, ecological traits can be rapidly measured from just one or a few isolates to inform pathogen risk prioritisation at an early stage while impacts can still be prevented.

Here, we develop a risk prioritisation model by predicting the global patterns of new detections (since 2005) of species of *Phytophthora* de Bary (‘plant‐destroyer’), a diverse genus of oomycete plant pathogens infecting a broad range of woody and herbaceous hosts in multiple sectors, with a history of disease spread among agricultural, horticultural, urban, commercial forestry and native woodland settings (Grünwald *et al*., 2012; Jung *et al*., 2016; Burgess *et al*., 2017; Dale *et al*., 2022). *Phytophthora* species are widespread and diverse within horticultural supply chains in multiple regions (Hardy, 1988; Green *et al*., 2021; Weiland, 2021). Studies of population genetics implicate horticultural pathways in the global spread of several high‐impact species, including *Phytophthora multivora* (Tsykun *et al*., 2022), *Phytophthora cinnamomi* (Shakya *et al*., 2021), *Phytophthora ramorum* (Jung *et al*., 2021) and *Phytophthora plurivora* (Schoebel *et al*., 2014).

Understanding the interplay between global trade networks and biological traits in driving pathogen invasion success is critical to identify pathogen species with greater capacity to exploit these pathways. Analysis of these factors helps to identify targets for preventative biosecurity interventions, including surveillance optimisation (Pasquali *et al*., 2015). Here, we compile a dataset of 1826 first detections of *Phytophthora* species comprising 181 species across 174 countries. We link new detections since 2005 to bilateral trade connectivity to source regions (pre‐2005), climate matching and plant health surveillance effort, using pathogen traits and phylogenetic position to infer species‐level differences in patterns of new detections and identify source regions with a higher risk of future invasions. Using a species‐level trait database including all *Phytophthora* species represented in our analysis (Marcot *et al*., 2023), we test the hypotheses that broader thermal tolerance ranges and dormancy adaptations (presence of chalmydospores and/or oospores) enhance the effect of trade connectivity, surveillance effort and climatic similarity. We discuss the potential impacts of our models for horizon scanning and interventions to mitigate plant pathogen transport through trade.

## Description

We tested the effects of two traits (thermal tolerance range and oospores/chlamydospores) and three potential risk factors for new detections of *Phytophthora* species since 2005:

1. The sum of horticultural imports (including fruit, vegetables, live plants, bulbs, roots and cut flowers) from all source countries where the *Phytophthora* species was known to be present before 2005 (transport risk).
2. Climate matching between the importing country averaged across all known source regions (all exporting countries where the *Phytophthora* species was present before 2005), within urban, agricultural and forested areas only (establishment risk).
3. The total number of records relating to each importing country in the European Plant Protection Organisation (EPPO) reporting service archives (surveillance effort).

These metrics integrate risks across known source regions and reflect this uncertainty in the model estimates. We do not model unique country combinations separately due to the paucity of information about global *Phytophthora* diversity and specific origins of newly detected *Phytophthora*, which precludes the attribution of arrival to different exporting countries for most *Phytophthora* species.

### *Phytophthora* new detection data

We collated a global database of *Phytophthora* species first reports (year) at the country level based on records from CAB Abstracts, CABI Invasive Species Compendium datasheets (CAB International, 2022), the European Plant Protection Organisation (EPPO) Global Database (EPPO, 2022), Global Biodiversity Information Facility (GBIF, 2022), National Center for Biotechnology Information (NCBI) BioSample (Sayers *et al.*, 2024) and Index Fungorum (Index Fungorum Partnership, 2022) as well as from a global network of pathologists and National Plant Protection Organisations (NPPOs). Our dataset includes detections in trade (nurseries, garden centres and ports‐of‐entry), amenity planting, gardens, urban areas, forestry and woodlands. However, as this information is not available for all *Phytophthora* records, we do not attempt to differentiate establishment in urban and natural environments. We also do not restrict the analysis to *Phytophthora* species that have established – species intercepted in trade are also included. First reports before the year 2005 were used to derive a binary matrix of the presence (1) or absence (0) of *Phytophthora* species (columns) in each country (rows), representing the known source distribution of each species. First reports after 2005 were used to derive a binary matrix of absence (0) and new detections (1) of *Phytophthora* species (columns) in each country (rows) in the year 2005 or after, excluding countries where they were already found before 2005. We elected not to model first record years as there is substantial uncertainty around *Phytophthora* first record dates, with potentially long time lags between arrival and detection (e.g. > 20 yr for Ash dieback: Combes *et al*., 2024), compounded by lack of information about whether species are native or non‐native that makes it difficult to assign source and recipient regions correctly. We assume that time lags to detection have shortened since the availability of molecular diagnostic tools and therefore focus on new detections since 2005. Recent detections are also more closely aligned with the time period for which standardised, comprehensive and reliable commodity‐level reporting of trade data are available in Resource Trade Earth (since 2000).

For some *Phytophthora* species, population genetic studies have identified their likely source regions. For example, the native origins of *Phytophthora lateralis* (Brasier *et al*., 2010), *P. ramorum* (Jung *et al*., 2021) and *P. multivora* (Tsykun *et al*., 2022) have been traced back to populations in Taiwan, Japan and South Africa, respectively. Where this published information is not captured in our data sources, we have adjusted the source regions manually. We acknowledge that the source regions of *Phytophthora* species will not be fully captured in this dataset and may disproportionately capture already invaded countries where impacts have been more severe. Primary invasions from native source regions are likely to be under‐represented, especially where high population diversity has yet to be detected in potential source regions. Additionally, for countries with little historical survey effort and for species with poorly documented ranges, it is possible that some of the newer detections of *Phytophthora* could be within parts of their native range. Our data do not distinguish native and non‐native ranges of *Phytophthora* because this is not widely recorded in our global data sources. Any *Phytophthora* occurrence reported before 2005 (whether native or non‐native) is considered a potential source for subsequent *Phytophthora* invasions (e.g. Grünwald *et al*., 2012; Tsykun *et al*., 2022), and new detections since 2005 may represent either native or non‐native species.

### Trade data

Annual trade in horticultural commodities (live plants, roots, bulbs and cut flowers) between importing and exporting country pairs was extracted from Resource Trade Earth (Chatham House, 2022) for the years 2000–2004 in units of 1000 kg. This represents a snapshot of trading patterns immediately before our set of new *Phytophthora* detections (2005–2022) and an attempt to account for the expected time lags between transport and first reports (Seebens *et al*., 2015). These data were used to derive a matrix of mean annual trade flows in these commodities between exporting countries (rows) and importing countries (columns) during this time. To capture transport risk for each species‐country combination, we derived a proxy for relative propagule pressure as the sum of imports of live plants from all exporting countries where the *Phytophthora* species was present before 2005 (e.g. from the potential source regions). Horticultural trade volumes were log‐transformed to improve normality for modelling.

### Climate‐matching data

Climatic distance between exporting and importing countries was based on four variables. Minimum temperature of the coldest month (bio6), mean temperature of the warmest quarter (bio10) and precipitation seasonality (bio15) were extracted from WorldClim2 (Fick & Hijmans, 2017), and potential evapotranspiration (Global‐ET0) was used to capture soil moisture (Trabucco & Zomer, 2019). Cold stress is a limiting factor in the distributions of *Phytophthora* species (Redondo *et al*., 2015; Burgess *et al*., 2019), and the minimum temperatures for growth in our trait database are positively correlated with the values of bio6 and bio10 at occurrences of those species (Green *et al*., 2021). Seasonality of precipitation is important for the completion of the life cycle of *Phytophthora* species: Germination of *Phytophthora* reproductive structures is triggered by moisture and warm conditions, and the infective propagules of *Phytophthora* are transmitted through surface water. Dry stress parameters in CLIMEX models have been found to limit the global distributions of *Phytophthora* species (Ireland *et al*., 2013; Burgess *et al*., 2017). To account for the association of *Phytophthora* species with horticultural, agricultural and forest host plants, we also calculated spatial layers of the percentage of forest, agriculture and urban cover using the Landcover CCI (European Space Agency, 2017). All climate and land‐use layers were reprojected to the Mollweide Equal Area projection and resampled to a resolution of 4500 m. The climate cells were then masked to retain only those cells with > 70% cover of urban, agricultural or forest land‐use types. The climate variables for the land‐use masked cells in each country were extracted using country polygons downloaded from Natural Earth Admin 0 – Countries (Natural Earth, 2022). A pairwise matrix of climatic distance metrics (and their SD) between exporting countries (rows) and importing countries (columns) was calculated using the Mahalanobis distance metric (Mahalanobis, 1936). This statistical metric measures how distant a point is from the centre of a multivariate normal distribution and has been used to measure multivariate environmental distances for species distribution modelling (Etherington, 2021). The climate matching metric for each exporter–importer combination was derived from the mean of the Mahalanobis distances from a single cell in the importing country across all cells in the exporting country, followed by taking the mean and SD of these values across all cells in the importing country. The Mahalanobis distances for each unique species‐country combination were square‐root‐transformed to improve normality for modelling. To express climate distance as a risk factor (e.g. as climate similarity, with areas at higher risk of invasion if they were more similar to source regions in climate), the variable was multiplied by −1.

### Phytosanitary surveillance data

We incorporate a measure of national recording effort in importing countries, specific to plant health, to account for the patterns of new *Phytophthora* detections arising from differences in surveillance and reporting of plant pathogens. Wealthier countries with larger plant health surveillance and research expenditures will contribute disproportionately to the new detections modelled in our analyses. To capture the engagement and activity of the NPPOs, we used the number of records per country in the EPPO Reporting Service cumulative index. Data were available for 212 countries with a median of 12 reports per country, a maximum of 812 and a minimum of 1 during the reporting period 1967–2022. The EPPO Reporting Service is a monthly newsletter collating events of phytosanitary concern, including new geographical records, new host plants and new diagnostic tools and methods (EPPO, 2022). Together, these reports represent a historical record of plant health reporting. It is acknowledged that these data may be biased towards regulated and emerging pests posing potential risks to the EPPO region, but the database is the most comprehensive historical archive of global plant health reporting publicly available. The number of reports were log‐transformed.

### *Phytophthora* trait and phylogenetic data

We used a species‐level trait database derived from *Phytophthora* species descriptions and other published literature (Marcot *et al*., 2023). Each species has a single trait value, reflecting the mean value where data were available for multiple isolates. We selected two traits to include in the analysis of *Phytophthora* new detections – thermal tolerance range and number of survival structure types. Temperature growth curves define the minimum, optimum and maximum thermal tolerances of pathogen isolates in laboratory‐controlled conditions. We selected the temperature range (maximum–minimum temperature for growth) to capture interspecific differences in the ability of *Phytophthora* species to survive fluctuating and extreme conditions during transport and to tolerate seasonality and climate differences between potential source regions and importing countries (Redondo *et al*., 2018; Barwell *et al*., 2021). Oospores and chlamydospores are dormant reproductive structures produced sexually and asexually, respectively. They have been hypothesised to persist, potentially for long periods, in soil (Fichtner *et al*., 2009) or host plant tissue (Crone *et al*., 2013b; Jung *et al*., 2013). Their presence will facilitate survival in the absence of a suitable host or environmental conditions and/or enable asymptomatic infections (Crone *et al*., 2013a) that increase the likelihood of evading early detection via visual inspections of live plants within live plant trade networks. The production of both asexual and sexual types of resting spores should enhance undetected spread, even in the absence of both mating types. The number of years since the *Phytophthora* species was described was used as a species‐specific metric of expected knowledge accumulated about distribution, spread and impacts. We use a seven‐locus phylogeny for *Phytophthora*, which includes all 109 *Phytophthora* species modelled (Bourret *et al*., 2023).

### Statistical model

All variables were centred and scaled by two SD to improve model convergence and to enable the comparison of effect sizes between binary and continuous predictors (Gelman, 2008). We fit Bayesian zero‐inflated generalised linear mixed effect models, adapted from a hierarchical model structure originally designed for trait‐based joint species distribution modelling (Pollock *et al*., 2012). This allows the intercept and slope parameters to vary among *Phytophthora* species to account for species variability in overall prevalence globally (variable intercepts) and the sensitivity of their global spread patterns to trade flows, climate matching and surveillance (variable slopes). We modelled new detections (1) and nondetections (0) of *Phytophthora* species *i*, in country *j*, as a zero‐inflated binomial (ZIB) response. The ZIB distribution is a mixture of a binomial distribution and a distribution with point mass of one at zero. It is specified as follows:

$$Y_{\mathrm{ij}}\sim \left\{\begin{array}{c} 0\;\text{with probability}\;p_{\mathrm{ij}}\;\\ Ɓ\;\left(m_{\mathrm{ij}},\pi_{\mathrm{ij}}\right)\;\text{with probability}\;1-p_{\mathrm{ij}} \end{array}\right.$$

where $p_{\mathrm{ij}}$ is a mixing probability to accommodate extra zeros (zero‐inflation) arising from a separate process or processes, and $Ɓ\;\left(m_{\mathrm{ij}},\pi_{\mathrm{ij}}\right)$ is the binomial distribution with trial size $m_{\mathrm{ij}}$ = 1 and event probability $\pi_{\mathrm{ij}}$. Both the event $\pi_{\mathrm{ij}}$, and mixing $p_{\mathrm{ij}}$, probabilities are modelled via logistic regression.

$$p_{\mathrm{ij}}={\text{logit}}^{-1}\left(z+\gamma_{k}⨯{\text{Detection}}_{\mathrm{ij}}\right)$$

To account for inflated nondetection due to country‐ and species‐level detectability among species, we modelled zero‐inflation $p_{\mathrm{ij}}$, as a function of a constant, *z*, across all observations adjusted for the effect of two detection variables ($\gamma_{k}⨯{\text{Detection}}_{\mathrm{ij}}$), one measured at country‐level (EPPO reporting service activity) and one measured at species‐level (the number of years since a species had been described):

The linear model of event probabilities $\pi_{\mathrm{ij}}$ is modelled as a crossed random effects model

$$\pi_{\mathrm{ij}}=\begin{array}{l} {\text{logit}}^{-1}(a_{\left[i\right]}+\alpha_{\left[i\right]}+e_{\left[j\right]}+\beta_{k\left[i\right]}⨯{\text{Risk factors}}_{\mathrm{jk}}\\ +\;\beta_{t}⨯{\text{Traits}}_{t}) \end{array}$$

where the logit probability that a species *i* is detected in country *j* of 1–56 countries is equal to the sum of the species‐ and country‐level intercepts *a*_[*i*]_, *α*_[*i*]_ and *e*_[*j*]_ plus the product of a matrix of countries by each of the *k* = 1, 2 or 3 risk factors for new *Phytophthora* detections since 2005 (${\text{Risk factors}}_{\mathrm{jk}}$) and a vector of their three coefficients $\beta_{k\left[i\right]}$. The final term is $\beta_{t}⨯{\text{Traits}}_{t}$, which is the product of a species by trait matrix and a vector of the trait coefficients. The square brackets indicate that there is a different parameter $\beta_{k}$ and different intercept terms, *a* and *α*, for each of the *i* ^th^ species in the set of 109 species

$$a_{i}\sim \text{Normal}\left(\mu_{a},\sigma_{a}^{2}\right)$$

where parameter *μ* is the estimated average global emergence risk (e.g. the proportion of countries with a new detection for that species on the logit scale) for an ‘average’ species with mean trait values in a country with mean values for all country‐level risk factors. The variance $\sigma_{a}^{2}$ represents the variability among observed species in their divergence from this global mean emergence risk.

We account for phylogenetic nonindependence among the species‐level observations using a unique species‐level intercept, $\alpha_{i}$, which follows a multivariate distribution with a mean of zero and between species variance $\sigma_{\alpha }^{2}$ due to the phylogeny, derived from the $N_{\text{species}}⨯N_{\text{species}}$ phylogenetic correlation matrix A.

$$\alpha_{\left[i\right]}\sim \text{Normal}\left(0,\sigma_{\alpha }^{2}A\right)$$

There are also different intercept terms for each of the *j* countries:

$$e_{\left[j\right]}\sim \text{Normal}\left(\mu_{e},\sigma_{e}^{2}\right)$$

where $\mu_{e}$ can be interpreted as the average invasion pressure (expected proportion of *Phytophthora* species recently detected per country on the logit scale) for a country with mean values of the risk factors and a species with mean trait values. This random effect structure allows us to partition the variance in new detections attributable to unmeasured species phylogenetic relationships and unmeasured species‐level and country‐level covariates and account for this uncertainty in model predictions.

Following Pollock *et al*. (2012), species sensitivity to each connectivity risk factor is allowed to vary by modelling partial regression slopes as

$$\beta_{k\left[i\right]}=C_{k\left[i\right]}+D_{\mathrm{kt}}⨯{\text{Traits}}_{t\left[i\right]}$$

We can interpret *C*_*k*[*i*]_ as a quantification of how much species differ from the predicted responses to each connectivity risk factor given the species set of trait values. These species‐level differences from the average responses to the different risk factors are normally distributed.

$$C_{k\left[i\right]}\sim \text{Gaussian}\left(U_{\left[k\right]},\eta_{\left[k\right]}\right)$$

with means $U_{\left[k\right]}$ and SD $\eta_{\left[k\right]}$ for each of the *k* = 1, 2, or 3 connectivity risk factors for introduction. The terms *U*_[*k*]_ are the average species‐level difference from the expected response to the risk factors given the species trait values and $\eta_{\left[k\right]}$ is the overall species‐level variability. The term $D_{\mathrm{kt}}$ is the matrix of coefficients for each risk factor and ${\text{Traits}}_{t\left[i\right]}$ is a matrix of trait values for each of the two ecological traits (number of survival structures and thermal tolerance range). Including these interaction terms between species traits and trade connectivity, climate matching and surveillance allows us to estimate with $D_{\mathrm{kt}}$ how much of the overall species‐level variation in responses to these risk factors is trait‐mediated. We test for four trait‐mediated responses: the number of survival structures and their effect on (1) sensitivity to trade connectivity and (2) surveillance effort, and the thermal tolerance range of species and its effect on the sensitivity of species recent spread to (3) trade connectivity and (4) climate matching with source regions.

### Model comparison

We implemented the models in the R package brms (Bürkner, 2017). To examine the relative importance of the different risk factors for new *Phytophthora* detections, we fitted 178 alternative models comprising all possible subsets of three risk factors for new detections (horticultural trade with source regions, climate matching with source regions and plant health surveillance) and three species‐level predictors (thermal tolerance range, number of survival structures and years since described, the latter as a proxy for accumulated knowledge about a pathogen), comprising 2^6^ = 64 models. Where both a trait and risk factor were present in the model, an additional model, also including the interaction term, was fitted (114 models) to test whether the different responses of pathogens to each risk factor were partially dependent on pathogen traits.

Model selection was based on the efficient approximate leave‐one‐out cross‐validation information criterion (Vehtari *et al*., 2017) to identify the most informative models about new *Phytophthora* detections, balancing explanatory power against predictive capacity for unseen data points. By comparing model performance using all combinations and subsets of these three different risk factors, with and without their interactions with trait values, we tested for a consistent and robust effect of trait‐mediated filtering of *Phytophthora* propagules, and if present, whether this is strongest during transport (greatest sensitivity to trade) or establishment (greatest sensitivity to climate matching) stages of an invasion or linked to detection probability (more sensitivity to plant health surveillance effort).

We provide an example of using the modelling framework to predict the top 20 current and anticipated *Phytophthora* threats to a target country, the United Kingdom. The ranking may be subsequently tailored to different regions or sectors by coupling with metrics relevant to specific host lists or habitats. These estimates of risk of new detections since 2005 can be produced for any country included in the analysis to inform national or sector‐specific pest prioritisation tools, for example the UK Plant Health Risk Register.

## Results

### Recent global patterns of *Phytophthora* new detections

Of the 182 *Phytophthora* species with first detection dates, 69 (38%) were either unknown to science before 2005 or no previous source region was known, precluding their use in the bilateral modelling framework. Data on bilateral horticultural trade flows, climate matching and plant health surveillance efforts were available for 135 countries. We retained only the 56 countries with at least two new detections since 2005 because a minimum level of replication within random effect factors (e.g. country) improves convergence of hierarchical models and should also reduce error arising from including countries with no history of reporting *Phytophthora* detections (potential false absences). After these exclusions, the data comprised 5309 species‐country observations comprising 1242 detections of 109 *Phytophthora* species since 2005 across 56 countries. *Phytophthora* species were newly detected in 0–20 countries, with a median of three countries per species. Countries reported between 2 and 30 new *Phytophthora* species detections since 2005, with a median of 6 per country. Data on *Phytophthora* new detections were biased towards Europe, which accounted for 23 of the 56 countries in the analysis, but countries from all five continents were represented in the model (Supporting Information Results S1), capturing regional variation in horticultural trading patterns, climate and plant health surveillance, with low multicollinearity between covariates (Results S2).

### Model performance

We identified 13 best‐performing models within two information criterion units of the best‐ranked model (Results S3). We report the median point estimates for variance explained and effect sizes and, in brackets, the lower and upper 95% credible intervals. Across these best‐performing models, the risk factors and pathogen traits, together with phylogenetically structured and species‐ and country‐level errors, explained between 72.4% (95% credible interval 52.0, 83.1) and 78.0% (61.8, 84.6) of variance in the probability that a species was newly detected in a country since 2005. The partitioning of the total variance explained among the model components is described in Table 1.

**Table 1:** Partitioning of percentage variance in new *Phytophthora* species detections explained by different model components.

Model component	Description	Percentage variance explained (95% credible interval range)
Fixed effects	Invasion risk factors (horticultural imports from source regions, climate matching, surveillance, pathogen traits and trait–risk factor interactions)	30.7 (24.9, 36.0) to 32.7 (25.4, 38.6)
Random intercepts	Residual variance in *Phytophthora* species global prevalence	10.1 (5.6, 16.3) to 11.5 (6.9, 17.8)
	Residual variance in importing country new reports	4.6 (2.8, 7.8) and 5.0 (2.9, 8.3)
	*Phytophthora* phylogenetic signal	3.3 (0.0, 16.8) and 9.6 (1.6, 24.6)
Random slopes	*Phytophthora* species variation in responses to trade connectivity	1.2 (0.0, 7.7) and 2.2 (0.0, 9.7)
	*Phytophthora* species variation in responses to climate matching	1.6 (0.0, 6.6) and 2.7 (0.0, 9.0)
	*Phytophthora* species variation in responses to surveillance	12.3 (4.9, 21.6) and 15.7 (7.8, 25.8)

The two values reported for each model component reflect the range (minimum and maximum variance explained) across the 13 best‐performing models. Bayesian models provide a posterior distribution of estimates. We report the median of this distribution and, in brackets, the 95% credible interval range.

One or more interaction terms between species‐level traits and risk factors for new detection were present in nine of the 13 best performing models. The parameter estimates for nondetection (zero‐inflation), invasion risk factors, traits and trait‐mediated effects were broadly similar across the top‐ranked models in which they were included (Results S3). For comparison and to reflect the full uncertainty from alternative candidate models with similar support based on cross‐validation predictions, we present the average parameter estimates across the 13 best‐performing models.

### Global patterns of nondetection

**Fig. 1:**
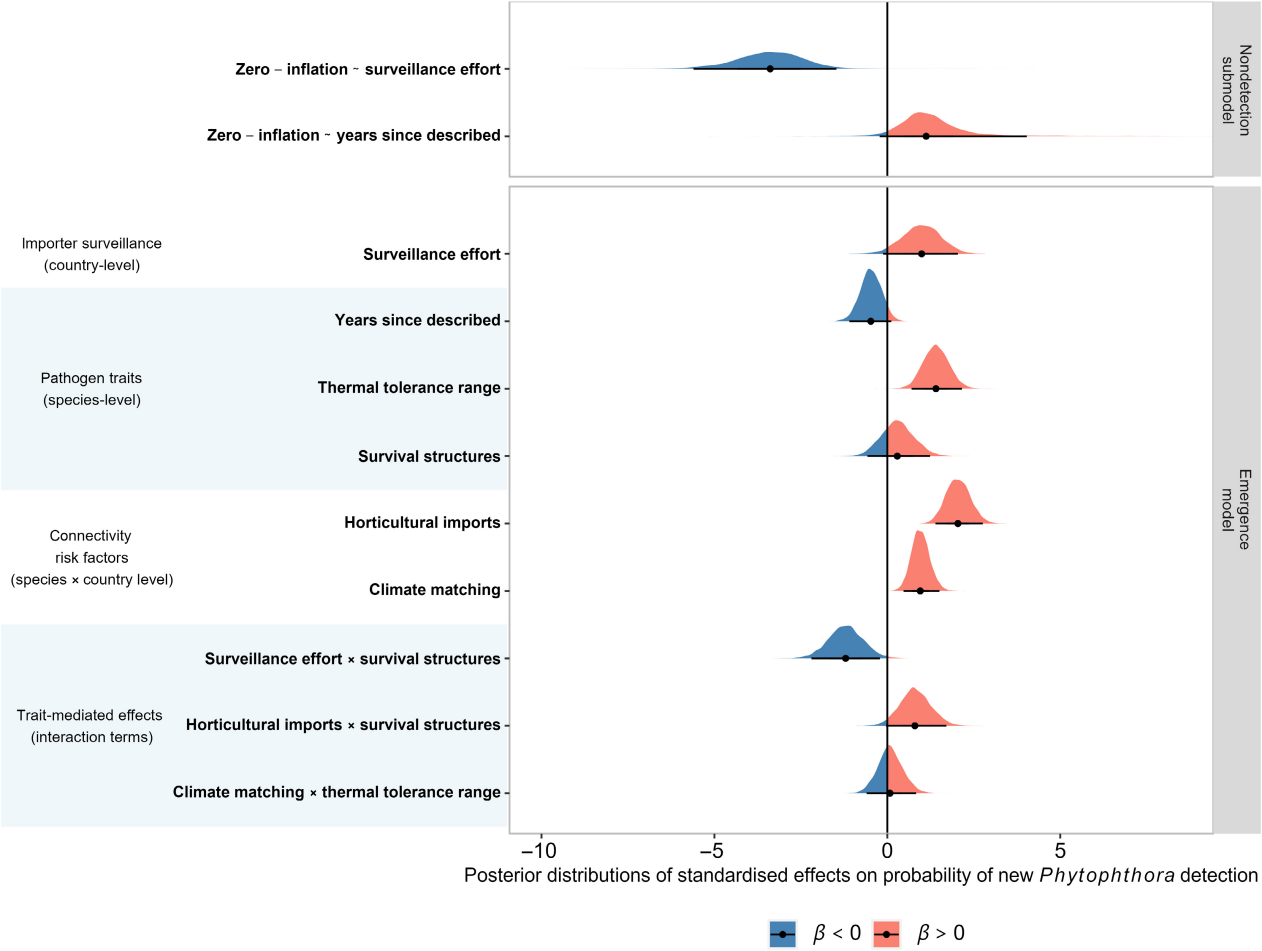
Relative importance of risk factors for new detections of *Phytophthora* species since 2005. Density plots are the posterior distributions for each parameter estimate, *β*, averaged across the 13 best‐performing models within two information criterion units (using approximate leave‐one‐out cross‐validation) of the best model out of 178 candidate models. Red and blue shading shows the portions of the posterior distributions for the parameter estimates that are greater (red) or less than (blue) zero (vertical black line representing no effect). Black dots show the median estimate and black slabs show the 95% credible intervals.

On average, across all potential species‐country combinations, the probability of a new *Phytophthora* detection since 2005 was estimated at 0.022 (95% credible = 0.003, 0.110). Nondetections of pathogens in importing countries where they are present can arise due to the genuine absence of the *Phytophthora* species or a lack of national‐level plant health reporting and general under‐recording of lesser‐known *Phytophthora* species. We attempted to disentangle the distinct processes of invasion and detection by modelling zero‐inflation within the detection data in relation to country‐level plant health surveillance effort and species‐level knowledge (proxied by national reporting activity and time since the pathogen species was described, respectively). Our model has both a detection submodel (probability of discovery) and a process model (probability of invasion). The zero‐inflation portion of the model describes the probability of an excess 0, which may estimate failure to detect *Phytophthora* species that are present. On average, globally, the probability of excess zeros was estimated at 0.15 (0.01, 0.39) among the 109 described *Phytophthora* species with known source regions. Odds ratios from the best‐performing model indicate that a 10% increase in national reporting activity reduced the odds of nondetection by a factor of 72.6% (58.8, 87.7). On average, an increase in historical plant health surveillance activity from 26 (25^th^ percentile) to 161 (75^th^ percentile) reports reduced the predicted probability of failing to detect a *Phytophthora* species presence from 0.39 (0.05, 0.66) to 0.06 (0.002, 0.22), indicating that, in a country with 26 historical EPPO reports, 39% of the 109 modelled *Phytophthora* species present may be missed, while a country with 161 historical EPPO reports would be expected to miss only 6% of globally described *Phytophthora* species that were present. Even after accounting for the effect of surveillance in reducing excess zeros, there was still a remaining positive effect of surveillance on the probability of a new detection since 2005 (Fig. 1), implying that the zero‐inflation model is not fully accounting for false absences.

Pathogen knowledge had a weaker effect on nondetection: A 10% increase in the number of years since a pathogen was described increased the odds of nondetection by 111.5% (96.3, 152.3) (Fig. 1; Results S3). On average, an increase in years since a species was described from 12 (25^th^ percentile) to 46 (75^th^ percentile) increased the probability of nondetection from 0.14 (0.007, 0.36) to 0.31 (0.03, 0.63), indicating that new detections since 2005 are more often of species that have been more recently described.

### Global drivers of *Phytophthora* detections since 2005

Our best‐performing models supported a role for all three risk factors (trade connectivity with source regions, climate matching with source regions and importer surveillance) and pathogen thermal tolerance range in driving global patterns of *Phytophthora* emergence (Fig. 1). The three risk factors were present in all 13 best‐performing models, while pathogen thermal tolerance range was present in seven of the 13 top‐ranked models, including the top model. Pathogens were more likely to be detected in countries with greater horticultural imports from potential source regions. A 10% increase in horticultural imports (after transforming and scaling) increased the odds of a new detection by 122.0% (114.3, 130.1). An increase in horticultural trade flows from 446 (25^th^ percentile) to 114 397 (75^th^ percentile) metric tons increased the probability of a new detection from 0.01 (0.0001, 0.417) to 0.04 (0.0005, 0.63). To a lesser extent, climate matching with the potential source regions also increased the risk of new species detections since 2005. A 10% increase in climate matching increased the odds of new detection by 109.7% (104.8, 115.5). An increase in Mahalanobis climate distance from 6.26 (25^th^ percentile) to 10.5 (75^th^ percentile) decreased the probability of a new detection from 0.02 (0.0002, 0.049) to 0.01 (0.0001, 0.49). Recent detections were also predicted to be more frequent in countries with a history of greater plant health surveillance. A 10% increase in historical national EPPO reporting activity increased the odds of a recent detection by 110.9% (100.1, 123.0). After accounting for the effect of surveillance on nondetection, an increase in historical EPPO reporting activity from 26 (25^th^ percentile) to 161 (75^th^ percentile) reports increased the probability of a new detection from 0.01 (0.0001, 0.35) to 0.03 (0.0005, 0.58). Of the two traits we tested, the models only supported a main effect of thermal tolerance range on the patterns of new detections globally. Species with broader thermal tolerance ranges emerged more often than species with narrower thermal limits. A 10% increase in thermal tolerance range increased the odds of a new detection globally by a factor of 114.2% (106.9%, 122.8). In absolute terms, an increase in species thermal tolerance range from 22°C (25^th^ percentile) to 28°C (75^th^ percentile) increased the probability of a new detection from 0.01 (0.0001, 0.39) to 0.02 (0.0003, 0.55).

### Species‐level variation in responses to risk factors

**Fig. 2:**
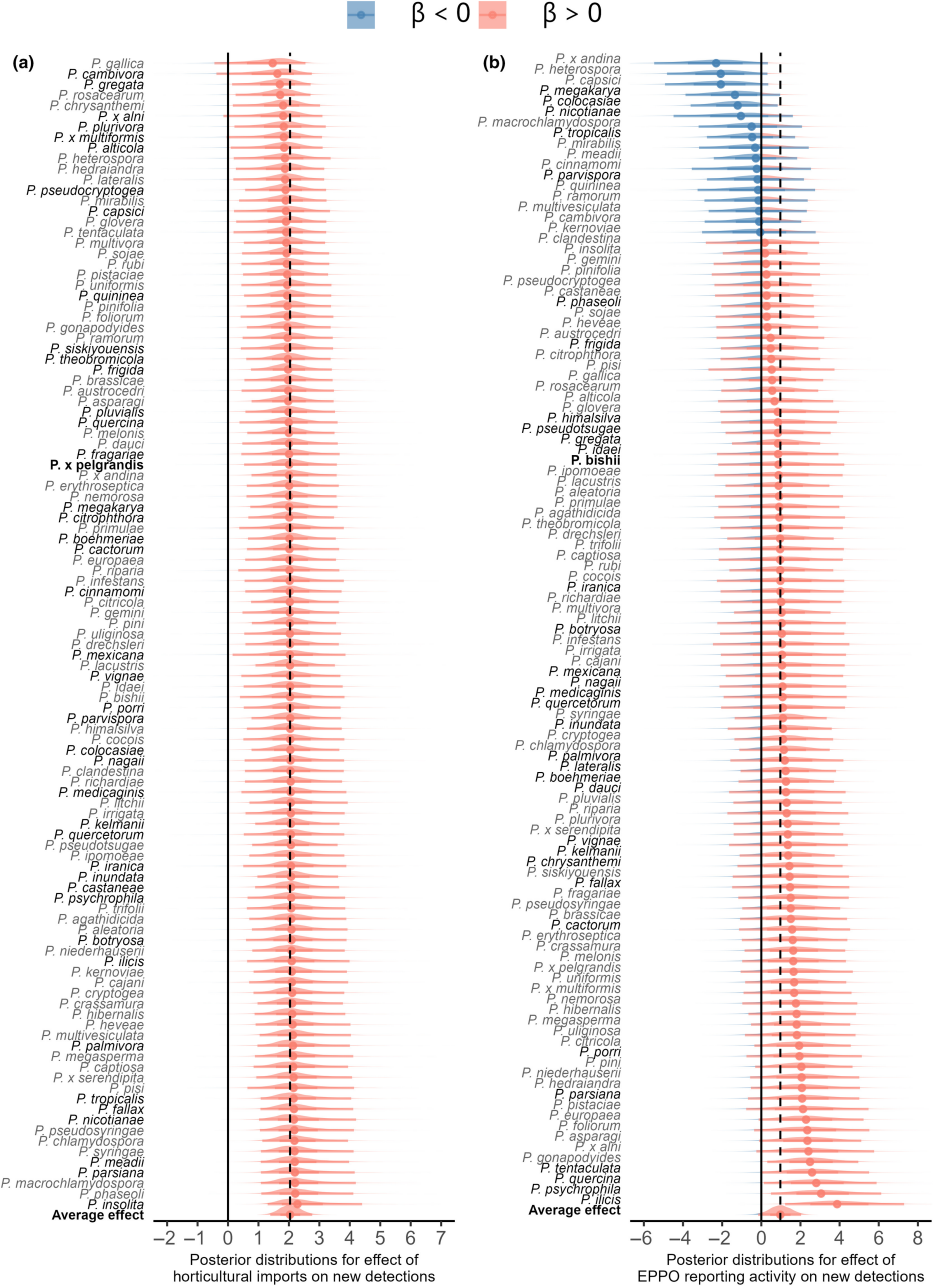
Species‐level responses to risk factors for invasion. Variation in *Phytophthora* species sensitivity to (a) horticultural imports and (b) European Plant Protection Organisation reporting activity (a proxy for country‐level surveillance effort) and the average effect across 109 *Phytophthora* species (vertical dotted lines). Species are ordered from weaker or more negative responses (top) to strongly positive responses (bottom) to trade or surveillance and vary in the magnitude and sometimes direction of their responses. Red indicates positive effect sizes greater than zero (solid vertical line) and blue indicates negative effect sizes. Solid vertical lines represent an effect size of 0. Points show the median estimate and slabs show the 95% credible intervals of the effect size for each species. Species in bold text are those producing both types of survival structures (chlamydospores and oospores). Species did not vary significantly in responses to climate matching (not shown).

Using species‐level random slopes, we estimated species partial responses for each risk factor to quantify differences in sensitivity of *Phytophthora* species detections to these different risk factors and the contributions of pathogen species traits to this variability (Fig. 2). New detections for all species responded positively to horticultural trade connectivity and climate matching with the species source region, but with some variability among species (Fig. 2a). We detected the strongest species‐level differences in responses to surveillance effort, in which both the strength and sign of the effect were predicted to differ depending on the focal species (Fig. 2b). We report species‐level effects of a 10% increase in surveillance effort, along with the 95% credible intervals, which account for uncertainty arising from species‐ and country‐level random effects. A 10% increase in historical national EPPO reporting activity decreased the odds of a new detection to 82.8% (62.1, 106.1) for the species with the most negative response to surveillance (*P*. × *andina*) but increased the odds of detection to 145.3% (113.4, 200.0) for the species with the strongest positive response to surveillance (*Phytophthora ilicis*). An increase from 25 (25^th^ percentile) to 160 (75^th^ percentile) historical reports increased the probability of a new detection of *P. ilicis* from 0.001 (0.00, 0.03) to 0.03 (0.002, 0.27), but decreased the predicted probability of a new detection of *P*. × *andina* from 0.27 (0.03, 0.63) to 0.13 (0.01, 0.61). A 10% increase in trade connectivity increased odds of a new detection by 115.0% (93.4, 125.0) to 124.5% (112.8, 155.9) depending on the focal species (Fig. 2a). An increase in trade connectivity from 446 to 114 397 metric tons increased the probability of a new detection for *Phytophthora insolita* from 0.02 (0.002, 0.22) to 0.13 (0.01, 0.46), while for *Phytophthora gallica*, the probability of a new detection increased from 0.16 (0.02, 0.59) to 0.32 (0.02, 0.77). A 10% increase in climate matching increased the odds of a new detection of *Phytophthora heterospora* and *Phytophthora quercina* by 103% (87.83, 114.2) and 115.6% (105.0, 138.6), respectively.

### Trait‐mediated sensitivity of pathogens to risk factors

**Fig. 3:**
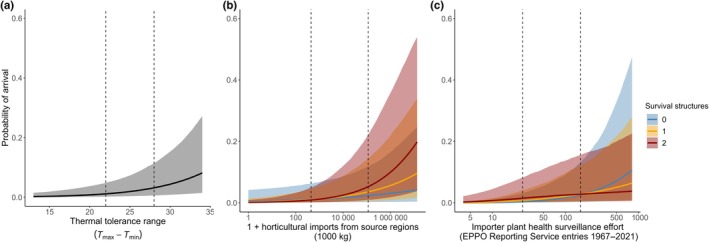
Role of pathogen traits and trait‐mediated effects in predicting global patterns of *Phytophthora* new detections since 2005. Marginal effects of thermal tolerance ranges of *Phytophthora* species (a), trait‐mediated effects of horticultural imports (b) and plant health surveillance effort (c) on the probability of a new detection since 2005, depending on the number of dormant survival structures (0 = neither oospores nor chlamydospores; 1 = oospores OR chlamydospores; 2 = oospores AND chlamydospores). Vertical lines illustrate the portions of the slopes (25^th^ and 75^th^ percentile of the scaled covariate values) used to report the absolute effect sizes and strength of interactions in the text. Shaded areas show the 95% credible intervals of the marginal effects.

Two pathogen traits (number of survival structures and thermal tolerance range) were used to test for trait‐mediated effects of global risk factors for new detections of *Phytophthora*. The functional value of these traits at the introduction stage was tested through their interaction with horticultural trade connectivity and plant health surveillance effort, while interactions with climate matching were interpreted as evidence of traits promoting establishment. While greater thermal tolerance range increased the overall risk of a new detection, only the pathogen number of survival structures mediated pathogen species responses to global connectivity risk factors (Fig. 3). We report the magnitude of these significant interaction terms as both the odds ratio (relative odds) and additive difference measures (differences in absolute risk) between the 25^th^ and 75^th^ percentile of trade connectivity, climate matching and surveillance effort to account for the potential influence of modelling scale on the interpretation of the statistical interactions in binary logistic models (Spake *et al*., 2023).

We detected a trait‐mediated response to horticultural trade connectivity, mostly evident at higher trade volumes (Fig. 3b). The interaction term between the number of survival structures and trade connectivity was present in six of the 13 best‐performing models. For a 10% increase in horticultural imports from known pathogen source regions, each additional survival structure increased the odds of a new detection by 107.9% (99.3, 117.6). In absolute terms, increasing horticultural trade connectivity from 446 (25^th^ percentile) to 114 397 metric tons (75^th^ percentile) increased the probability of a new detection among species with no survival structures from 0.01 (0.00, 0.49) to 0.02 (0.00, 0.61). Among species with one or two types of survival structure, the same increase in trade connectivity led to a greater estimated increase in detection probability from 0.01 (0.00, 0.45) to 0.04 (0.00, 0.65) and 0.01 (0.00, 0.43) to 0.06 (0.00, 0.70), for the 25^th^ and 75^th^ percentiles, respectively.

The interaction term between the number of survival structures and surveillance effort was present in six of the 13 best‐performing models. The effect of plant health surveillance on recent global spread was weaker for pathogens producing one or more dormant survival structures (Fig. 3c). For a 10% increase in surveillance effort, each additional survival structure reduced the odds of a new detection by 89.12%. For pathogens without survival structures, an increase in surveillance from 25 (25^th^ percentile) to 160 (75^th^ percentile) historical reports increased the probability of a new detection from 0.003 (0.00, 0.25) to 0.03 (0.0002, 0.60), but for species with one or two survival structures, there was a weaker increase in detection probability from 0.007 (0.00, 0.34) to 0.03 (0.00, 0.61) and from 0.02 (0.00, 0.45) to 0.03 (0.00, 0.65), respectively.

### Predicted global patterns of *Phytophthora* risks

**Fig. 4:**
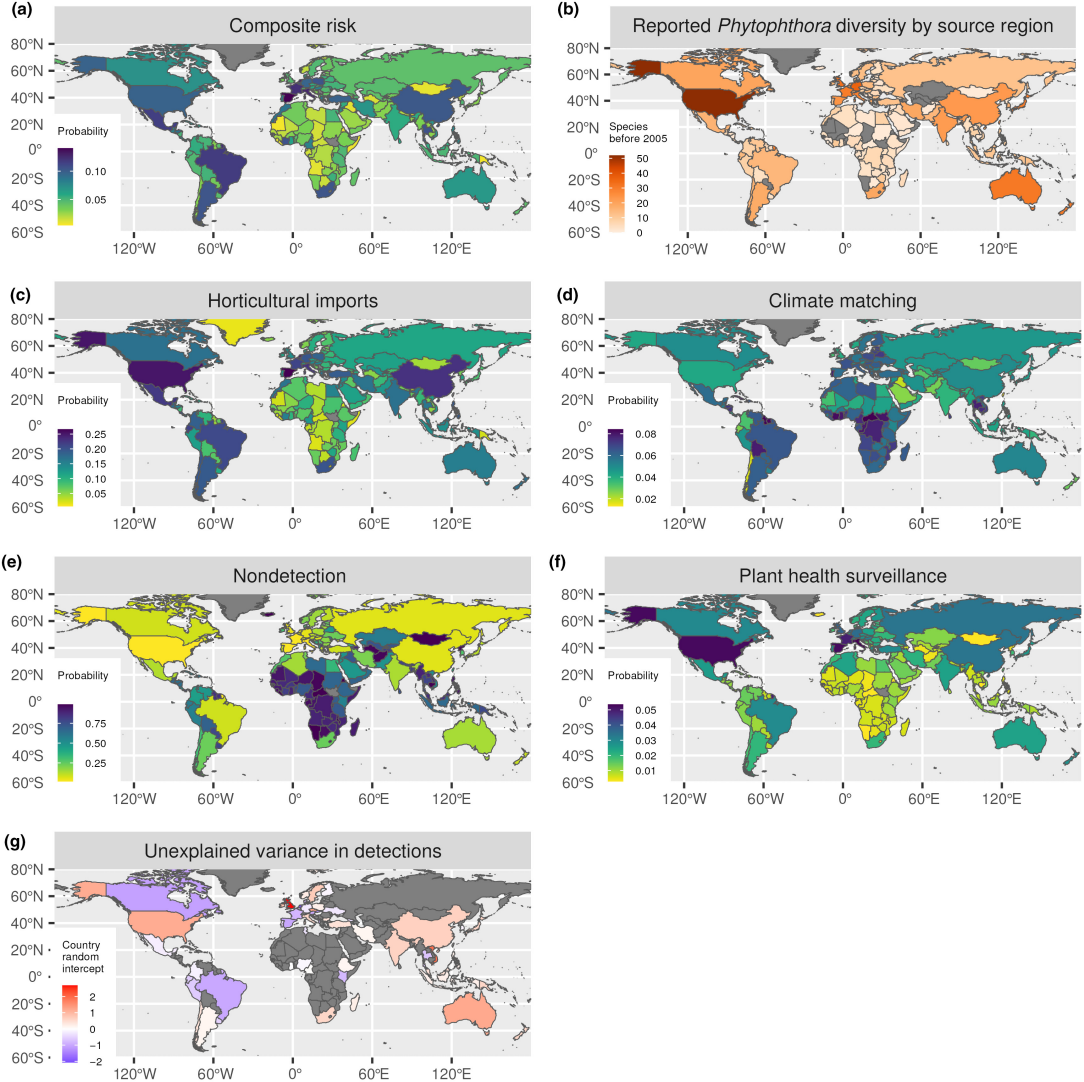
Drivers of new *Phytophthora* detections since 2005. Predicted risk of a new *Phytophthora* species detection presented as (a) composite risk combining horticultural imports from source regions, climate matching with source regions, plant health surveillance effort, pathogen traits and phylogenetic position. Reported *Phytophthora* diversity (b) before 2005 used to define source regions for potential invasions. Marginal effects (predicted risks when fixing all other variables at their mean) of horticultural imports from source regions (c), climate matching with source regions (d) and plant health surveillance effort (d) on the probability of a new detection since 2005. The zero‐inflation submodel predicts the probability of nondetection (e) using metrics of species‐level (not shown) and country‐level surveillance effort (f). Higher levels of nondetection imply greater uncertainty in predictions of composite risk for these regions. Random intercepts estimate the variance among countries in detection probability that is not explained by the risk factors of horticultural imports, climate matching and plant health surveillance (g).

**Fig. 5:**
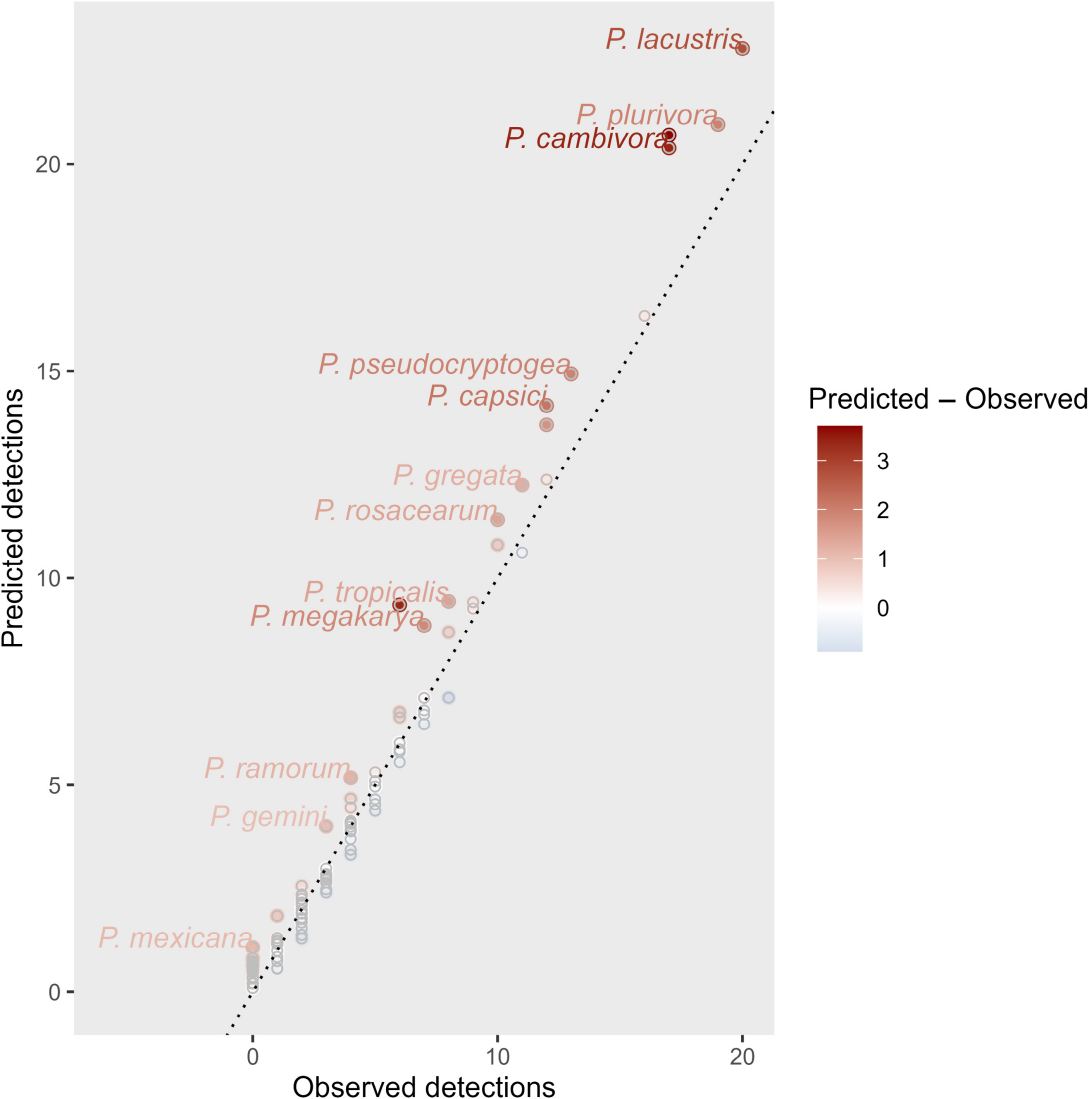
Relationship between predicted and observed numbers of detections for 109 *Phytophthora* species. The fill colour of points represents the magnitude of deviation from the observed number of detections, with darker red colours representing the species whose numbers of detections are much lower than would be predicted based on patterns of trade connectivity to source regions, climate matching with source regions and pathogen traits. Examples of underpredicted species are labelled and represent species that may be more widespread globally than are currently documented. Predictions are adjusted to account for country‐level differences in surveillance effort.

We estimate country‐level *Phytophthora* risks by combining the risk factors of trade connectivity and climate matching with potential source regions, historical surveillance effort and pathogen traits (Fig. 4; Notes S1), incorporating species‐level and country‐level variability arising from unmeasured covariates in the credible intervals. Regions where new *Phytophthora* species are most at risk of being introduced or are likely to already be present in greater numbers than have been detected to date include Spain, France and Italy in Europe, and Brazil, Argentina and Mexico in South and Central America. In Africa, South Africa and Côte d'Ivoire were predicted to be at the highest risk of new *Phytophthora* detections. China and Thailand were also highlighted as potential hotspots for new detections (Fig. 4a). High risks of *Phytophthora* introductions are driven predominantly by high levels of horticultural trade connectivity and climate matching with the source regions of *Phytophthora* yet to arrive (Fig. 4c,d), although climate matching (Fig. 4d) and plant health surveillance activity (Fig. 4f) had weaker marginal effects on predicted invasion risk. There are substantial uncertainties in the predicted risks for different countries (Fig. 4g). Despite high levels of surveillance and low probability of nondetection (Fig. 4e,f), some European countries including France and Spain are still reporting fewer *Phytophthora* introductions than would be predicted based on the risk factors (Fig. 4g). For other regions such as the United Kingdom, Sweden, Vietnam and Australia, recent detections exceed the predicted risks (Fig. 4g), reflecting more extensive knowledge of *Phytophthora* diversity through recent published surveys of native and managed ecosystems in these regions (Fig. 4b). Species‐level predictions are compared with observed levels of detection to identify potentially under‐reported *Phytophthora* species: those with smaller global distributions than would be predicted based on trade connectivity and climate matching with their source regions and their traits (Fig. 5).

### Predicting high‐risk source regions

**Fig. 6:**
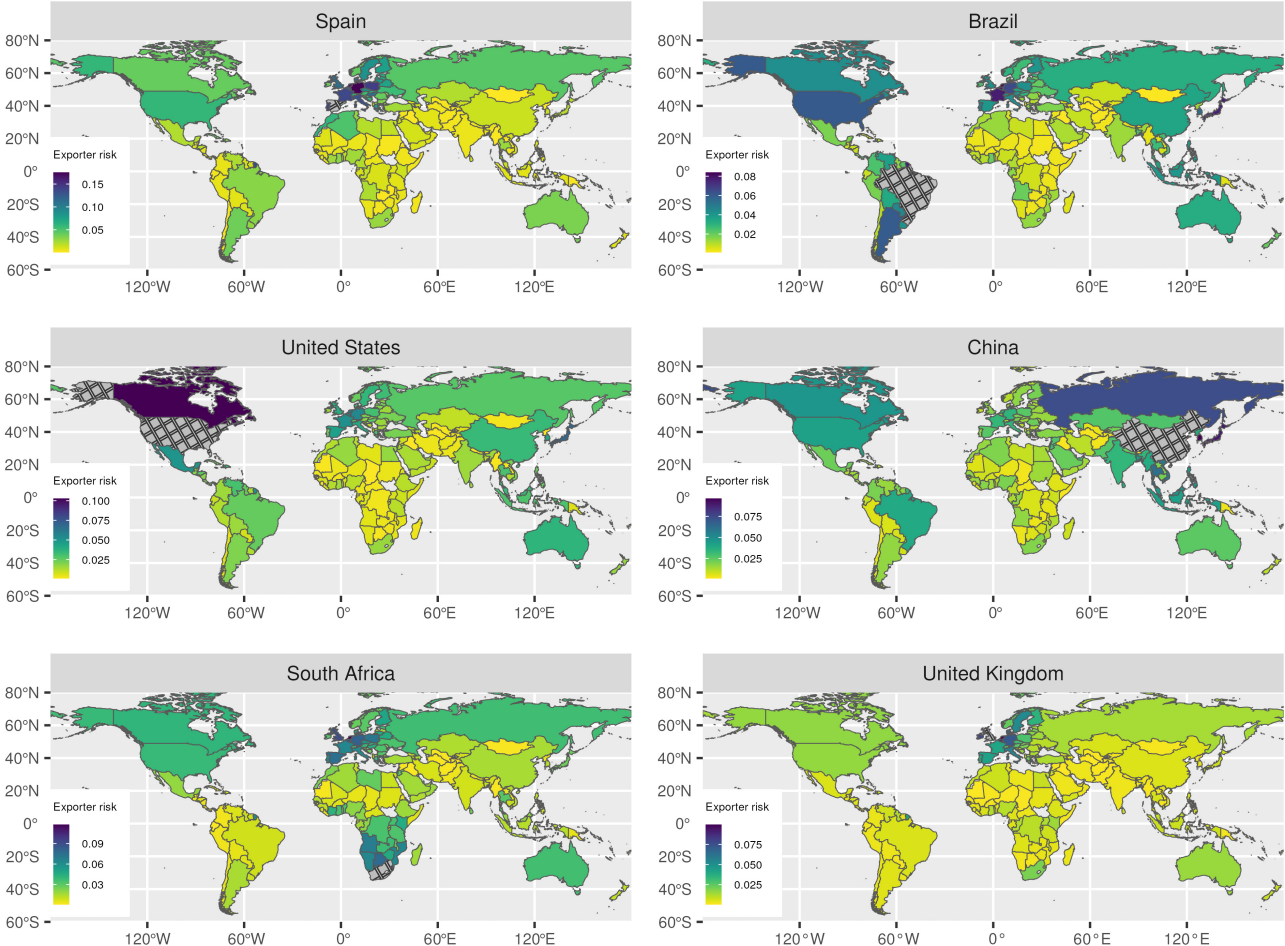
*Phytophthora* risks from different source regions. Predicted risk that exporting countries world‐wide will act as source regions for *Phytophthora* species for six example importing countries (Spain, Brazil, the United States, China, South Africa and the United Kingdom) with different global trading patterns, climatic regions and plant health surveillance effort. The six importing countries are ranked in order of their overall predicted risk of *Phytophthora* new detections since 2005 from highest (Spain) to lowest (United Kingdom). Importing countries are shown with grey hatching.

For a focal importing country, the United Kingdom, we identify high‐risk source regions for live plant imports and the likely geographic origins of recent new detections. The predictions for high‐risk source regions are based on trade flows and climate similarity with each exporting country, the known *Phytophthora* diversity within those regions, pathogen traits and phylogeny. For the United Kingdom (Fig. 6), neighbouring European countries are identified as key source areas for new *Phytophthora* introductions based on previous horticultural trade flows (2000–2005), climatic similarity and known *Phytophthora* diversity not yet reported in the United Kingdom. Outside Europe, the United States is predicted to pose the greatest risk of transporting novel *Phytophthora* species to the United Kingdom. The predictions can be tailored to any focal importing regions. We present examples of importation risk maps for six countries (Fig. 6) with differing levels of predicted *Phytophthora* emergence risk (see Fig. 4, for composite risk).

## Discussion

We demonstrate a modelling framework for predicting future threats from *Phytophthora* species in relation to the risk of transport through key pathways of international spread. We use the models to predict global patterns of *Phytophthora* invasions. Within countries, the predicted arrival risks and source regions can be coupled with metrics capturing, for example at‐risk hosts or habitats (Table 2) to assess and rank potential impacts.

**Table 2:** Example application of the modelling framework to rank and prioritise current and future *Phytophthora* threats to the United Kingdom using risk factors of trade with source regions, climate matching with source regions, plant health surveillance and pathogen traits.

	Predicted invasion risk (95% credible interval)	Global spread	Host genera	Lat. limits (decimal degrees)	UK detections in trade	UK detections in wider environment	NVC habitats at risk	On UK PHRR
**Current threats (present in the United Kingdom)**								
*Phytophthora pseudocryptogea*	0.87 (0.66, 0.97)	30	296	57.19	421	297	12	Yes
*Phytophthora rosacearum*	0.85 (0.60, 0.96)	14	21	52.93	1	0	8	No
*Phytophthora niederhauserii*	0.84 (0.58, 0.96)	19	55	51.33	3	3	4	No
*Phytophthora hedraiandra*	0.84 (0.56, 0.96)	20	17	54.04	0	14	5	No
*Phytophthora asparagi*	0.83 (0.54, 0.95)	12	11	41.23	na	na	0	No
*Phytophthora europaea*	0.77 (0.47, 0.94)	9	6	48.22	na	na	5	No
*Phytophthora psychrophila*	0.74 (0.39, 0.92)	10	29	56.05	4	0	8	No
*Phytophthora kelmanii*	0.74 (0.43, 0.91)	23	11	56.05	16	1	7	No
*Phytophthora capsici*	0.72 (0.32, 0.93)	61	18	55.94	0	1	6	No
*Phytophthora gregata*	0.72 (0.43, 0.91)	14	28	56.81	0	1	8	No
*Phytophthora pini*	0.70 (0.39, 0.90)	16	62	51.31	6	0	6	No
*Phytophthora tentaculata*	0.69 (0.37, 0.90)	14	19	51.51	1	2	9	Yes
*Phytophthora sojae*	0.69 (0.31, 0.90)	22	19	56.05	8	0	7	No
*Phytophthora gallica*	0.65 (0.32, 0.89)	15	9	60.05	0	1	10	No
*Phytophthora lateralis*	0.62 (0.26, 0.87)	23	44	56.42	0	3	4	No
*Phytophthora tropicalis*	0.62 (0.27, 0.86)	24	12	51.79	18	76	3	Yes
*Phytophthora brassicae*	0.58 (0.23, 0.85)	9	2	56.05	1	0	2	No
*Phytophthorauliginosa*	0.48 (0.15, 0.79)	3	6	57.19	8	0	6	No
*Phytophthora austrocedri*	0.37 (0.09, 0.72)	10	14	57.95	98	431	4	Yes
*Phytophthora riparia*	0.37 (0.08, 0.74)	4	5	60.68	4	0	0	No
**Anticipated threats (not yet detected in United Kingdom)**								
*Phytophthora gemini*	0.69 (0.31, 0.91)	4	1	No data	na	na	1	No
*Phytophthora crassamura*	0.67 (0.32, 0.90)	10	32	41.20	na	na	9	No
*Phytophthora parvispora*	0.62 (0.27, 0.88)	16	18	43.05	na	na	0	No
*Phytophthora frigida*	0.61 (0.28, 0.87)	5	3	−29.97	na	na	0	No
*Phytophthora* × *pelgrandis*	0.59 (0.22, 0.87)	5	5	53.23	1	0	1	No
*Phytophthora pinifolia*	0.44 (0.13, 0.80)	1	1	−37.29	na	na	3	No
*Phytophthora* × *serendipita*	0.44 (0.14, 0.81)	5	7	na	na	na	0	Yes
*Phytophthora heterospora*	0.42 (0.12, 0.79)	10	13	na	na	na	3	No
*Phytophthora multivesiculata*	0.38 (0.09, 0.73)	10	1	na	na	na	0	No
*Phytophthora alticola*	0.37 (0.07, 0.75)	1	1	−27.52	na	na	0	No
*Phytophthora megakarya*	0.34 (0.09, 0.68)	12	3	na	na	na	0	No
*Phytophthora parsiana*	0.33 (0.09, 0.69)	5	6	na	na	na	1	No
*Phytophthora colocasiae*	0.32 (0.07, 0.68)	37	12	23.40	na	na	0	No
*Phytophthora clandestina*	0.30 (0.06, 0.69)	1	1	na	na	na	1	No
*Phytophthora insolita*	0.29 (0.06, 0.65)	7	5	10.01	na	na	0	No
*Phytophthora chrysanthemi*	0.28 (0.06, 0.64)	7	1	45.94	na	na	0	No
*Phytophthora himalsilva*	0.28 (0.05, 0.67)	5	6	na	na	na	0	Yes
*Phytophthora meadii*	0.26 (0.06, 0.62)	28	27	10.07	na	na	0	No
*Phytophthora melonis*	0.26 (0.05, 0.61)	10	10	41.20	na	na	0	No
*Phytophthora pisi*	0.23 (0.02, 0.68)	1	na	na	na	na	0	No

The top 20 *Phytophthora* threats are ranked from highest to lowest based on predicted probability of repeated introductions (for species already present) or novel arrivals (for absent species). Greater risk reflects high‐trade connectivity and climate matching with known source regions and/or pathogen traits promoting spread. To support prioritisation of current and future threats on national risk registers like the UK Plant Health Risk Register (UK PHRR), predicted arrival risks can be coupled with metrics of potential impact including previous global spread (number of countries reported) host genera (number host genera reported), global latitudinal limits (Barwell *et al*., 2021), and the types of hosts and habitats at risk (e.g. by intersecting known global host species with National Vegetation Classification (NVC) Floristic Tables). na, no data available for the species.

Leveraging on existing species knowledge from global surveillance networks, our study suggests that around half of the variation in global patterns of new detections of *Phytophthora* species can be predicted. Incorporating species traits into predictions of invasion has significant potential to improve national and global horizon scanning and management of plant health risks. Some traits are already being integrated, via expert elicitation, into the European Food Safety Authority (EFSA) methodology for pest prioritisation (EFSA *et al*., 2019), but quantitative modelling to identify and validate the importance of key pathogen traits is lacking. Horticultural imports and climate matching with the source regions of *Phytophthora* species, along with metrics of national plant health surveillance efforts and pathogen traits, are key predictors of *Phytophthora* detections since 2005.

Even allowing for the diversity of unknown pathogen species, potential threats should be better anticipated by integrating across potential sources of uncertainty at the species level (representing phylogenetic and unmeasured traits, including undocumented source regions) and country level (representing unmeasured country‐level risk factors).

There remains some variation in country‐level detections not captured within our models (Fig. 4g), which may reflect the presence of expert research groups, or differences in preventative biosecurity or trading practices (e.g. quarantine facilities, trade bans or conversely free trade agreements). National reporting of horticultural imports does not account for the geographic origins of plants moved rapidly through multiple countries. Improved mapping of global horticultural supply chains would improve estimates of connectivity to specific source regions via major trade hubs.

We find that recently arrived *Phytophthora* species tend to have broader thermal tolerance ranges. This effect holds irrespective of patterns of trade connectivity and climate matching among the potential source and recipient countries. Species with narrow thermal tolerance ranges emerge less frequently in general, even in importing countries with high levels of horticultural trade connectivity and climatic similarity to the species source region. One explanation is that species with broader thermal tolerance ranges are likely to have greater global prevalence simply by tolerating a greater diversity of climates and environmental conditions. For example, cold‐tolerant *Phytophthora* species have been detected in more countries globally and have more extreme latitudinal limits (Barwell *et al*., 2021).

Another mechanism by which broad thermal tolerance may link to successful *Phytophthora* invasions is through rapid evolutionary change. Extreme, novel and variable conditions during transport and establishment potentially drive natural selection of increased thermal tolerance by filtering out *Phytophthora* propagules with narrow thermal tolerance ranges. There is limited evidence for rapid evolutionary change during invasions because trait comparisons between source and introduced populations are difficult to achieve when the source populations are typically unknown. Evolutionary lability in thermal traits has been detected in the *Phytophthora* genus (Chaloner *et al*., 2020; Notes S2), as well as in other invasive species, including seaweed (Sotka *et al*., 2018), plants (D. S. Chapman *et al*., 2017) and reptiles (Claunch *et al*., 2021).

On average, across all species, surveillance increased the probability of detection, but counter‐intuitively for a small number of species (e.g. *P*. × *andina*, *P*. *heterospora*, *P*. *capisci*, *P*. *megakarya*, *P*. *colocasiae* and *P. nicotianae*), greater surveillance effort was predicted to reduce rates of detection. This may be an artefact of unreliable slope estimates for rarer species (e.g. very few new detections globally since 2005). Alternatively, higher surveillance effort may be conflated with proactive biosecurity measures (not captured in our models) that may have prevented the introduction of these species. The global movement of these pathogens may also have occurred historically (before 2005) so that recent detections are relatively rare in countries with high levels of surveillance. This is consistent with our finding that new detections since 2005 are more often of species that have been recently described, perhaps already in their invaded regions, followed by rapid onward spread from those countries to others. The addition of such species to quarantine lists may enhance their rapid detection elsewhere.

We also find that new detections of species with one or both dormancy adaptations (oospores or chlamydospores) are more likely in countries with higher trade levels than species with neither or only one type of survival structure, suggesting these species can better exploit greater trade connectivity among source and recipient countries. Yet, countries with greater surveillance effort detect species with these dormancy adaptations less frequently than other species (Fig. 3c), despite their seeming affinity to trade pathways. Together, these findings support a potential role for dormancy adaptations in promoting the evasion of detection within traded plants. Traditionally, surveillance involves visual inspections of aerial parts of plants as a method of interception, yet these dormancy adaptations are found in soil and plant roots and are often associated with asymptomatic plants (Migliorini *et al*., 2015) as well as symptomatic plant tissue (Crone *et al*., 2013b) and also facilitate short‐ or long‐term survival within soil (McCarren *et al*., 2005; Fichtner *et al*., 2009; Gyeltshen *et al*., 2021). These findings strengthen the case for greater use of molecular tools within surveillance programmes, particularly high‐throughput meta‐barcoding pipelines, to enhance detection of unseen *Phytophthora* diversity being transported with live plants and/or soil (Green *et al*., 2021). Ensuring that inspections are not limited to the visual inspection of aerial parts of plants and including risk‐based or random screening of asymptomatic plants could help to improve the detection of species with these dormancy adaptations. However, we acknowledge that enhanced surveillance effort may be coupled with higher preventative biosecurity that may help to mitigate the pathways of spread with asymptomatic plants in these countries, especially in countries with greater levels of horticultural imports.

Other *Phytophthora* traits including lignitubers and stromata may also play a role in dormancy (Crone *et al*., 2013b), although they are not routinely reported in species descriptions. The role of different types of dormancy adaptations in promoting the avoidance of detection through visual inspections has yet to be confirmed through analysis of the species and morphological structures present in symptomatic and asymptomatic plants.

The models presented here will likely underestimate risks of primary invasions from native or unknown source regions, as we found that 38% of known *Phytophthora* species had unknown source regions and only a fraction of total *Phytophthora* diversity is estimated to have been formally described (Goheen & Frankel, 2009; Scott *et al*., 2019). Based on the predicted global patterns of nondetection, large areas of Africa, most of Southeastern Asia and Central and Southern America represent potential source pools of unknown *Phytophthora* diversity due to low surveillance capacity, high‐trade connectivity and diverse natural ecosystems. Risk analysis would benefit from greater investment in research and surveillance within natural and managed ecosystems in these regions, for example by establishing sentinel nurseries to identify the resident microbial diversity associated with frequently exported plants (Vettraino *et al*., 2017). Increasing numbers of surveys have been conducted in natural ecosystems in recent years, in some cases identifying the likely source regions for widespread, invasive *Phytophthora* species (Brasier *et al*., 2010; Jung *et al*., 2021, 2024), but the origins of nearly all invasive *Phytophthora* species are poorly understood. Unknown species are only partially accounted for in our models by integrating across these potential sources of uncertainty using random effects at the species and country level. Explicit integration of more informative proxies for the diversity of undescribed species in different source (exporting) regions may, potentially, improve our model predictions of future threats from *Phytophthora*, especially ‘unknown unknowns’ (e.g. measuring richness and diversity of plant species, diversity of crop production or export markets in different source regions). However, implementing these developments requires more comprehensive data on the origins of known *Phytophthora* species.

The models described here capture risk factors for new detections of *Phytophthora*. To support risk prioritisation, our predictions must be coupled with additional risk frameworks addressing multiple invasion stages, including establishment, spread and impact (EFSA *et al*., 2022). For example, linking pest outbreaks in the wider environment to the locations of high‐risk premise types or large‐scale planting activities can help to map the risk of introduction. Establishment risk can be assessed by linking pest outbreaks to spatial patterns of climate suitability, surveillance and mitigation. Integrating host susceptibility predictions with databases capturing host contributions to ecosystem service provision will enable prioritisation in pest management to be informed by values at risk, such as carbon sequestration, the cultural value of forests and other ecosystems as well as other metrics of impact (Gilioli *et al*., 2014). Pest and pathogen traits have the potential to inform predictions at each invasion stage and can be incorporated into risk frameworks (Barwell *et al*., 2023). There is a strong need to codevelop decision support tools from these risk frameworks with potential decision‐makers across sectors and scales to understand potential impacts on decision‐making and mitigation (Jones & Kleczkowski, 2020).

Applications may include prioritisation of inspections of consignments from high‐risk source regions, targeting these regions with sentinel plantings to improve knowledge of pathogen diversity and raising awareness among forestry and horticultural sectors to inform decisions about importing plants for planting. Additionally, ranking predicted invasion risks could support decisions about which pathogens to include on national risk registers. While trait‐based approaches are limited to known pathogens, the traits identified here are usually available within novel *Phytophthora* species descriptions, and their continued measurement is encouraged to support risk modelling frameworks like the one presented here. Extending this framework to include other pathogenic fungi and pests that share these horticultural transport pathways and methods of surveillance would provide a more comprehensive understanding of the traits that promote the successful transport of plant pests and diseases and the surveillance tools that are required to intercept their spread at an earlier stage.

## Competing interests

None declared.

## Author contributions

LJB, BVP and DC conceived the study. LJB developed the models and wrote the manuscript with contributions from all co‐authors. LJB, TIB and PS compiled distribution data. TIB, AP‐S, PS, NW, DELC, SG and TIB collated the trait data and provided pathology and evolutionary expertise to inform the trait‐based hypotheses and assist with the interpretation of results. All authors contributed critically to the drafts and gave approval for publication.

## Disclaimer

The New Phytologist Foundation remains neutral with regard to jurisdictional claims in maps and in any institutional affiliations.

## Supporting information


**Notes S1** Mapping risk factors for importing countries.
**Notes S2** Trait lability of focal *Phytophthora* traits.
**Results S1** Country‐level data on trade, climate matching and surveillance.
**Results S2** Multicollinearity between covariates.
**Results S3** Model comparison.
Please note: Wiley is not responsible for the content or functionality of any Supporting Information supplied by the authors. Any queries (other than missing material) should be directed to the *New Phytologist* Central Office.

## Data Availability

Data on new detections of *Phytophthora* since 2005 and the derived risk metrics are available in a public GitHub repository, along with the code to reproduce the model outputs presented at https://github.com/loubar/Phytophthora_trade_networks.
