# Supplementary material for: Trait‐mediated filtering of Phytophthora pathogen invasions through global horticultural trade networks
Source: New Phytol. 2025 Sep 19;248(5):2480–97. doi: 10.1111/nph.70587 (PMC12589713; doi:10.1111/nph.70587)
Supplement: Supplementary file 1 — Notes S1 Mapping risk factors for importing countries. Notes S2 Trait lability of focal Phytophthora traits. Results S1 Country‐level data on trade, climate matching and surveillance. Results S2 Multicollinearity between covariates. Results S3 Model comparison. Please note: Wiley is not responsible for the content or functionality of any Supporting Information supplied by the authors. Any queries (other than missing material) should be directed to the New Phytologist Central Office. [file NPH-248-2480-s001.pdf]

New Phytologist **Supporting Information**

Article title: Trait-mediated filtering of *Phytophthora* pathogen invasions through global horticultural trade networks

Authors: Louise J Barwell, Bethan V Purse, Sarah Green, Giles Hardy, Peter Scott, Nari Williams, David E L Cooke, Ana Perez-Sierra, Treena I Burgess and Daniel Chapman

Article acceptance date: 08 September 2025

The following Supporting Information is available for this article:

Results S1 Country-level data on trade, climate matching and surveillance

Results S2 Multicollinearity between covariates

Results S3 Model comparison

Notes S1 Trait lability of focal *Phytophthora* traits

Notes S2 Mapping risk factors for importing countries

Results S1 Country-level data on trade, climate matching and surveillance

Data on *Phytophthora* new detections were biased towards Europe, which accounted for half the 56 countries in the analysis, but countries from all five continents were represented in the model, capturing variation in horticultural trading patterns, climate and plant health surveillance among countries and global regions (Table S1).

Table S1 Geographical distribution of the 56 countries included in models of Phytophthora new detections

Continent	Number of countries included in model
Africa	8
Americas	10
Asia	12
Europe	23
Oceania	3

Results S2 Multicollinearity between covariates

Variation in trade connectivity to *Phytophthora* species' source regions varied by several orders of magnitude across country-*Phytophthora* pairs, while climate matching and plant health surveillance were less variable across countries and species (Fig. S1). Note that for modelling all covariates were centred to 0 and scaled by 2 standard deviations for comparability of effect sizes.

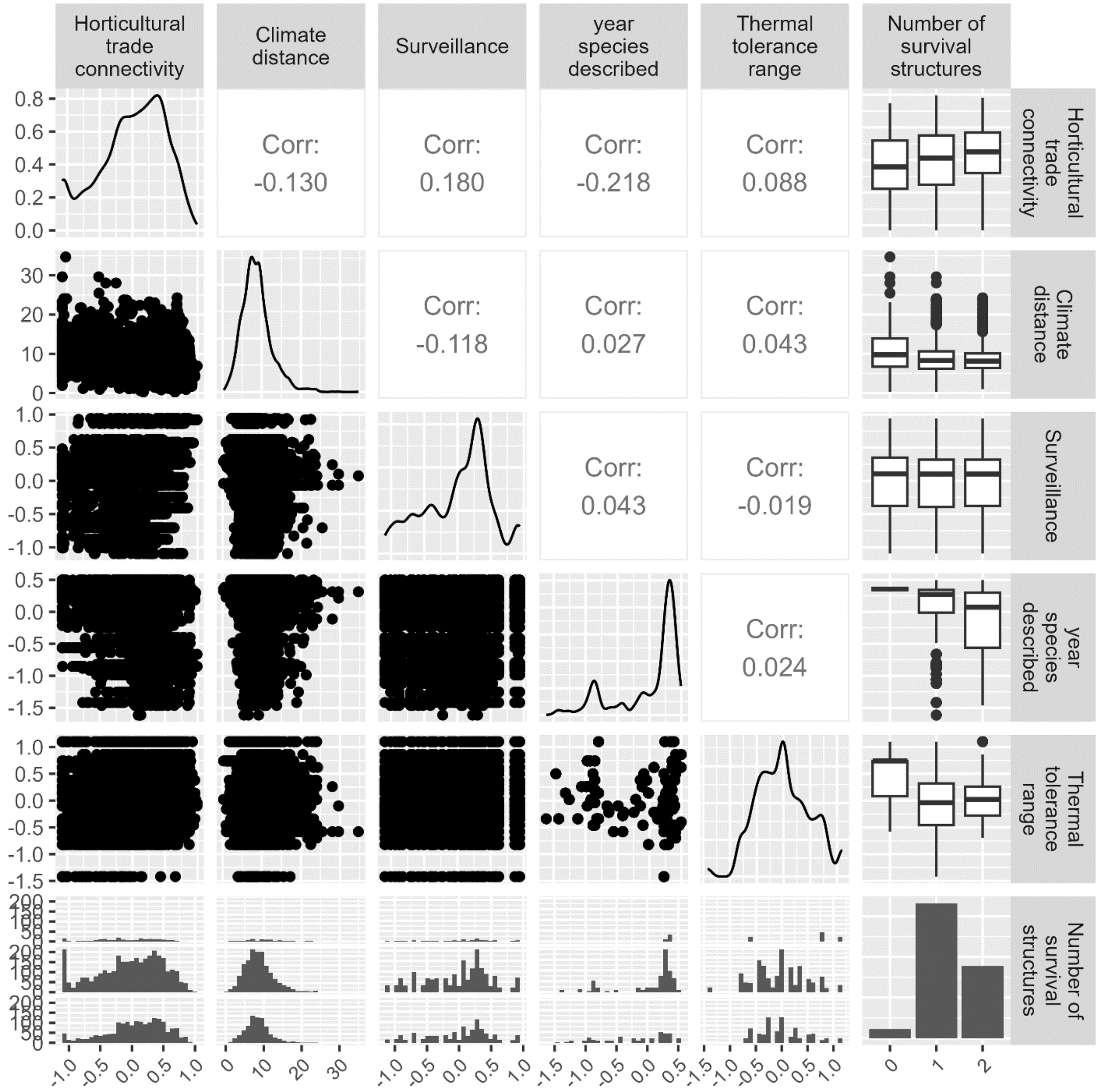

Figure S1 Distributions, pairwise scatterplots and Pearson correlation coefficients among covariates used as proxies for risk factors for Phytophthora arrival, across 5309 species-country pairs representing 56 countries and 72 Phytophthora species.

Multicollinearity between variables can mask effects of variables on the arrival risk by leading to low parameter estimates and high associated standard errors. We tested for collinearity between model variables using generalised variance inflation factors (VIF: Fox & Monette 1992) and found that all covariates showed low multicollinearity and were appropriate to include in the global model: All VIF values were close to 1, and well below the threshold of 3 at which multocollinarity may be considered problematic (Table S2).

Table S2 Variance Inflation Factors (VIF) and their 95% confidence intervals (lower CI) measuring multicollinearity between covariates included in the models of Phytophthora new detections. The adjusted VIF measures how much greater the standard error of the parameter estimates becomes due to the association with other predictors (conditional on other variables in the model).

Term	VIF	VIF lower CI	VIF upper CI	SE factor	Toleran ce	Tolerance CI low	Tolerance CI high
Survival structures	1.32	1.28	1.36	1.15	0.76	0.74	0.78
Climate similarity	1.06	1.04	1.09	1.03	0.95	0.92	0.96
Date described	1.09	1.07	1.13	1.05	0.91	0.89	0.94
Surveillance	1.08	1.06	1.12	1.04	0.92	0.89	0.94
Thermal tolerance range	1.07	1.04	1.1	1.03	0.94	0.91	0.96
Horticultural trade connectivity	1.12	1.09	1.15	1.06	0.89	0.87	0.92

Results S3 Model comparison

The best-performing models of *Phytophthora* new detections were identified through model selection based on efficient leave-one-out cross validation (Vehtari *et al*. 2017; Figure S1). There were 16 models with a difference in information criterion < 5 from the best-performing model which can be considered equivalent in performance (Fig. S2). Consistent parameter estimates across models indicates effects are robust to the inclusion and exclusion of other predictors in the models (Fig. S3).

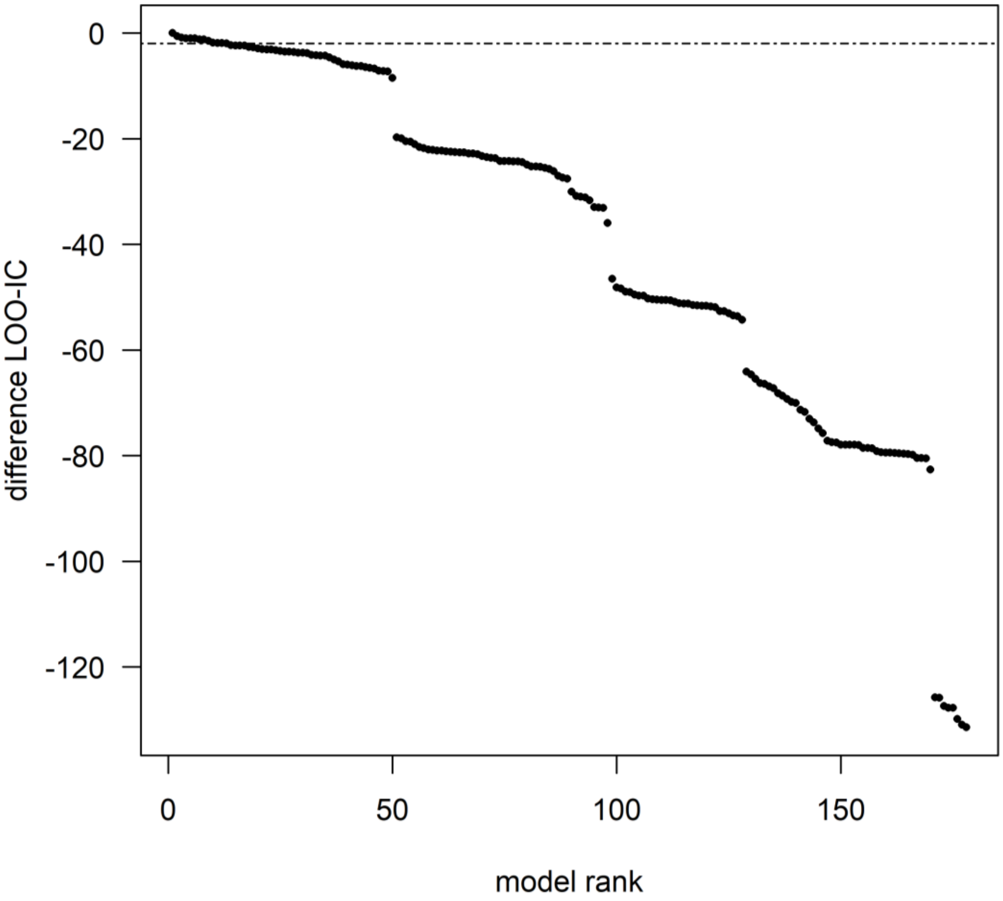

Figure S2 Difference in information criteria from the top-ranked model for all 178 candidate models of Phytophthora new detections since 2005

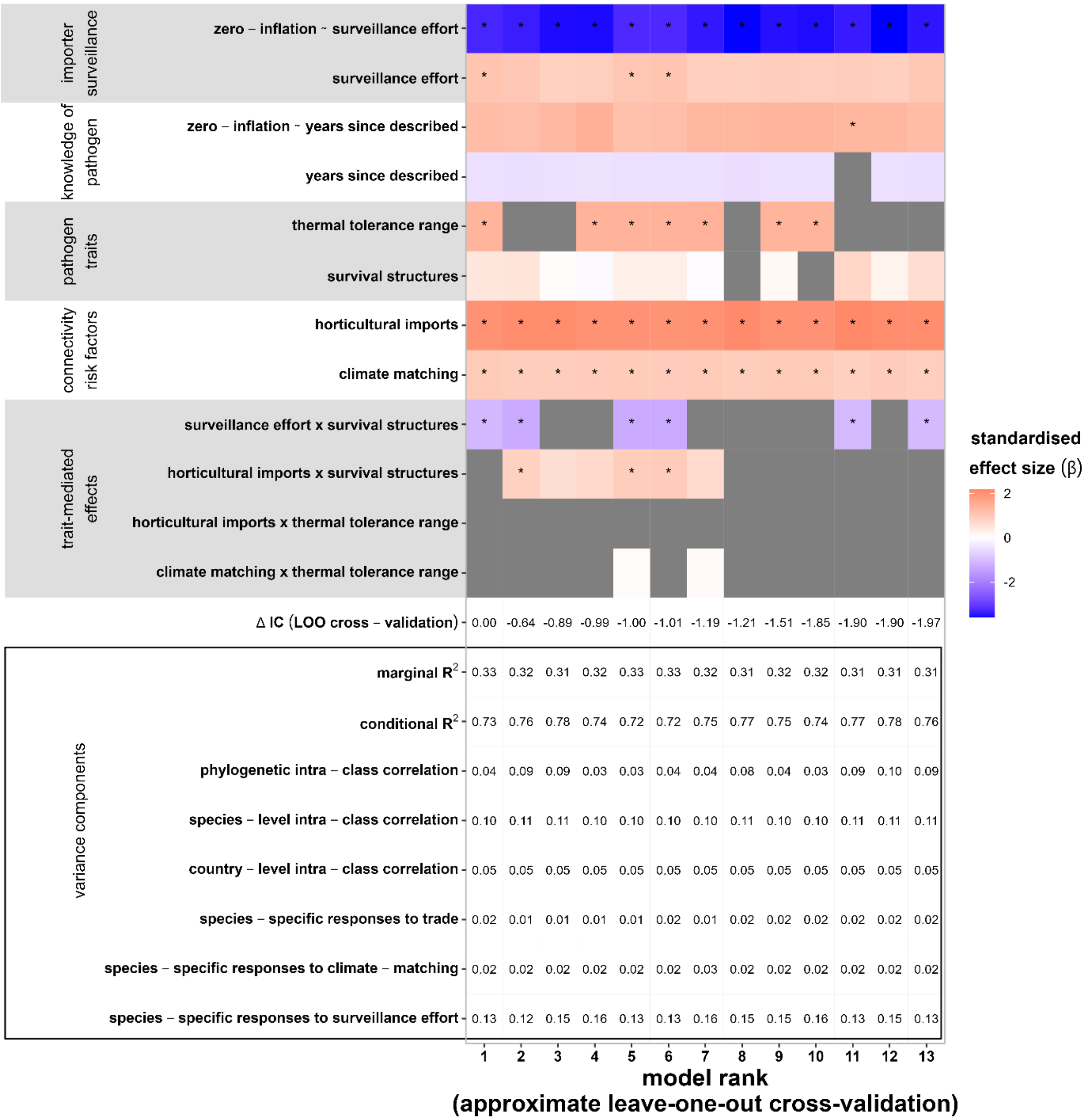

	1	2	3	4	5	6	7	8	9	10	11	12	13
Δ IC (LOO cross – validation)	0.00	-0.64	-0.89	-0.99	-1.00	-1.01	-1.19	-1.21	-1.51	-1.85	-1.90	-1.90	-1.97
marginal R^2	0.33	0.32	0.31	0.32	0.33	0.33	0.32	0.31	0.32	0.32	0.31	0.31	0.31
conditional R^2	0.73	0.76	0.78	0.74	0.72	0.72	0.75	0.77	0.75	0.74	0.77	0.78	0.76
phylogenetic intra – class correlation	0.04	0.09	0.09	0.03	0.03	0.04	0.04	0.08	0.04	0.03	0.09	0.10	0.09
species – level intra – class correlation	0.10	0.11	0.11	0.10	0.10	0.10	0.10	0.11	0.10	0.10	0.11	0.11	0.11
country – level intra – class correlation	0.05	0.05	0.05	0.05	0.05	0.05	0.05	0.05	0.05	0.05	0.05	0.05	0.05
species – specific responses to trade	0.02	0.01	0.01	0.01	0.01	0.02	0.01	0.02	0.02	0.02	0.02	0.02	0.02
species – specific responses to climate – matching	0.02	0.02	0.02	0.02	0.02	0.02	0.03	0.02	0.02	0.02	0.02	0.02	0.02
species – specific responses to surveillance effort	0.13	0.12	0.15	0.16	0.13	0.13	0.16	0.15	0.15	0.16	0.13	0.15	0.13

Figure S3 Standardised effect sizes in 13 best-performing models of Phytophthora species new detections since 2005. Models are ranked from left (top-ranked model) to right. Model parameters (fixed effects and interaction terms) are shown on the left. Significant effects (*) are parameter estimates with 95% credible intervals which do not overlap zero. Grey squares indicate the fixed effects were absent from the model. Red and blue colours indicate positive and negative parameter estimates, respectively, Deeper colours reflect stronger relative effect sizes. The random effects are quantified as the intra-class correlation (the variance explained in units comparable with the marginal and conditional R squared). Note that the variance components do not sum to the conditional R² because the estimates for the species-level intercepts and slopes are allowed to covary and are not independent.

Notes S1 Mapping risk factors for importing countries

Mapping the relative importance of the different risk factors on a common probability scale, as in Fig. 4 (main text) conceals some of the differences in the importance of individual risk factors vary among countries. We also map the individual risk factors using independent probability scales to allow for comparison of the effects of individual risk factors across different countries (Fig. S5).

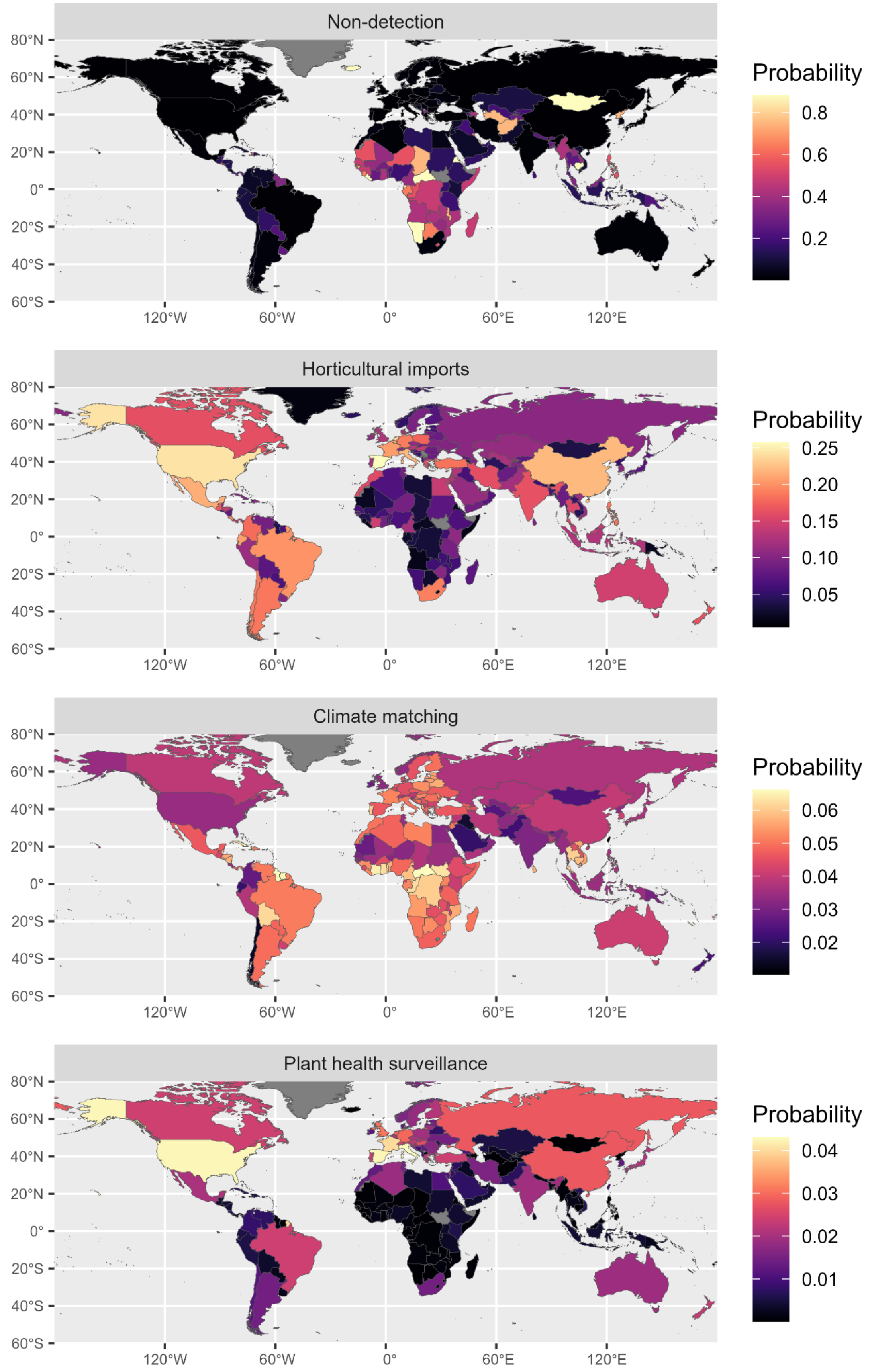

Figure S5 Marginal effects of risk factors for Phytophthora *new detections since 2005. Note that the predicted probability of a new detection is mapped using separate probability scales and therefore the maps do not represent the relative importance of different risk factors, but the variability between countries in the importance of the focal risk factor.*

Notes S2 Trait lability of focal Phytophthora traits

Disparity through time analyses compare morphological trait disparity within and across sub-clades (Harmon *et al*. 2003). The observed disparity is compared to the null hypothesis that trait values follow an uncorrelated random walk (Brownian Motion) along the branches of a phylogenetic tree. This can be mapped through time to explore the tempo of trait evolution. High levels of relative disparity indicate that sub-clades have independently evolved to have largely overlapping trait values across clades. If relative disparity is low relative to expectations under Brownian Motion then sub-clades have narrower and more distinct trait values. Disparity values significantly greater than the null suggest that subclades overlap, and all contain a significant proportion of variation found throughout the entire group at a given time. Negative disparity indicates that morphological variation is partitioned among subclades, indicating that each subclade occupies a distinct region of the morphospace. The Morphological Disparity Index (MDI) measures the area between the observed and expected patterns of morphological divergence. The MDI is positive for both *Phytophthora* temperature range (MDI = 0.25, P value between 0.0004 and 0.0164) and number of survival structures (MDI = 0.31, P value between 0.0004 and 0.020) and within clade disparity is significantly greater than expected under Brownian Motion based on the global rank envelope test for non-random trait diversification proposed by Murrell (2018). Therefore, the traits selected to inform the models of *Phytophthora* spread through trade networks are labile through evolutionary time, and especially so in more recent evolutionary history (Fig. S4, top panels). A key question is whether invasion drivers including trade and transport connectivity, climate change and biosecurity practice constitute natural selection pressures that are shaping the prevalence and evolution of favourable traits across the *Phytophthora* genus and promoting subsequent invasions.

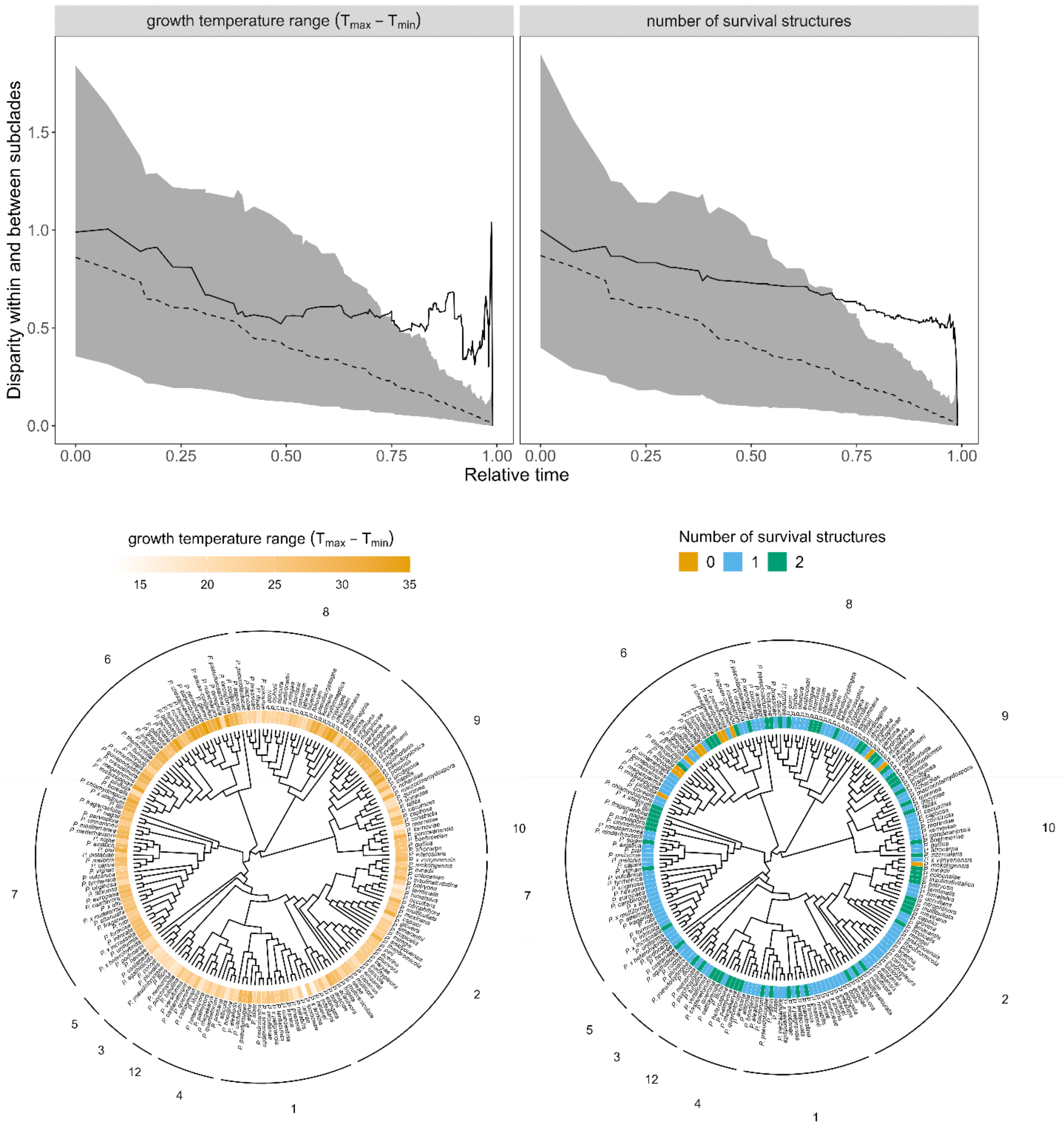

Figure S4 Disparity through time plots (top panels) for Phytophthora species in Clades 1 to 12. showing relative trait disparity within and between subclades at different points in relative evolutionary time, describing the node height in phylogenetic history from root (0) to tip (1). Solid black lines show observed trait disparity and dotted lines shows simulated trait disparity assuming an uncorrelated random walk (Brownian Motion) along the branches of a phylogenetic tree. The grey shaded area represents 95% confidence intervals for 2500 simulations under the null model. Where solid black lines fall outside of the grey shaded region, disparity is significantly different from null model expectations.

References

Fox, J., & Monette, G. (1992). Generalized Collinearity Diagnostics. Journal of the American Statistical Association, 87(417), 178–183.

Harmon LJ, JA Schulte, JB Losos, and A Larson. 2003. Tempo and mode of evolutionary radiation in iguanian lizards. *Science* 301:961-964. https://www.science.org/doi/10.1126/science.1084786
Murrell DJ. A global envelope test to detect non-random bursts of trait evolution. Methods Ecol Evol. 2018; 9: 1739–1748. https://doi.org/10.1111/2041-210X.13006
Vehtari, A., Gelman, A. & Gabry, J. Practical Bayesian model evaluation using leave-one-out cross-validation and WAIC. Stat Comput 27, 1413–1432 (2017). https://doi.org/10.1007/s11222-016-9696-4
